# Genomic Analyses and Transcriptional Profiles of the Glycoside Hydrolase Family 18 Genes of the Entomopathogenic Fungus *Metarhizium anisopliae*

**DOI:** 10.1371/journal.pone.0107864

**Published:** 2014-09-18

**Authors:** Ângela Junges, Juliano Tomazzoni Boldo, Bárbara Kunzler Souza, Rafael Lucas Muniz Guedes, Nicolau Sbaraini, Lívia Kmetzsch, Claudia Elizabeth Thompson, Charley Christian Staats, Luis Gonzaga Paula de Almeida, Ana Tereza Ribeiro de Vasconcelos, Marilene Henning Vainstein, Augusto Schrank

**Affiliations:** 1 Centro de Biotecnologia, Universidade Federal do Rio Grande do Sul, Porto Alegre, Brazil; 2 Laboratório Nacional de Computação Científica, Petrópolis, Brazil; University of California-Riverside, United States of America

## Abstract

Fungal chitin metabolism involves diverse processes such as metabolically active cell wall maintenance, basic nutrition, and different aspects of virulence. Chitinases are enzymes belonging to the glycoside hydrolase family 18 (GH18) and 19 (GH19) and are responsible for the hydrolysis of β-1,4-linkages in chitin. This linear homopolymer of N-acetyl-β-D-glucosamine is an essential constituent of fungal cell walls and arthropod exoskeletons. Several chitinases have been directly implicated in structural, morphogenetic, autolytic and nutritional activities of fungal cells. In the entomopathogen *Metarhizium anisopliae,* chitinases are also involved in virulence. Filamentous fungi genomes exhibit a higher number of chitinase-coding genes than bacteria or yeasts. The survey performed in the *M. anisopliae* genome has successfully identified 24 genes belonging to glycoside hydrolase family 18, including three previously experimentally determined chitinase-coding genes named *chit1*, *chi2* and *chi3*. These putative chitinases were classified based on domain organization and phylogenetic analysis into the previously described A, B and C chitinase subgroups, and into a new subgroup D. Moreover, three GH18 proteins could be classified as putative endo-*N*-acetyl-β-D-glucosaminidases, enzymes that are associated with deglycosylation and were therefore assigned to a new subgroup E. The transcriptional profile of the GH18 genes was evaluated by qPCR with RNA extracted from eight culture conditions, representing different stages of development or different nutritional states. The transcripts from the GH18 genes were detected in at least one of the different *M. anisopliae* developmental stages, thus validating the proposed genes. Moreover, not all members from the same chitinase subgroup presented equal patterns of transcript expression under the eight distinct conditions studied. The determination of *M. anisopliae* chitinases and ENGases and a more detailed study concerning the enzymes’ roles in morphological or nutritional functions will allow comprehensive insights into the chitinolytic potential of this highly infective entomopathogenic fungus.

## Introduction

Chitin is ubiquitous in nature and is synthesized by a wide range of organisms being considered surpassed in abundance only by cellulose [1], [2]. Chitin has an essential role in structuring biological composites, granting them structural shape and protective resistance. Chitin microfibrils, assembled by several N-acetyl-β-D-glucosamine subunits, serve as important structural constituents of cell walls from yeasts and filamentous fungi and exoskeleton from arthropods [1], [3], [4]. This linear homopolymer of N-acetyl-β-D-glucosamine can be hydrolyzed at β-1,4-linkages by the enzymatic action of glycoside hydrolase enzymes, the chitinases (E.C. 3.2.1.14) and the N-acetylglucosaminidases (E.C. 3.2.1.52). Chitinases are classified into two glycoside hydrolase families, GH18 and GH19, and N-acetylglucosaminidases belong to glycoside hydrolase family 20 (GH20) [5] (www.cazy.org). Both enzymes act in a synergistic and consecutive fashion to perform the complete enzymatic hydrolysis of chitin to N-acetylglucosamine (GlcNAc) [6]. Chitinases can also be classified by their mechanism of action. Generally, the endochitinases hydrolyze the chitin polymer at random internal chain points, while exochitinases release GlcNAc dimers (GlcNAc_2_) from the non-reducing end. However, a novel GH18 exochitinase was described that releases monomers from the reducing end by a processive mechanism [7]. The endo- and exo- mode of action can occur in combination with processivity. Processive enzymes do not release the substrate after a successful cleavage, but slide through the active site to promote the next cleavage [8], [9]. N-acetylglucosaminidases can act weakly on non-reducing ends of chitooligomers, but show preference for acting on diacetylchitobiose (GlcNAc_2_), producing GlcNAc monosaccharides [3].

The two glycoside hydrolase families, GH18 and GH19, contain chitinases which display differences on amino acid sequences, conserved domains and 3D protein structures [10]. The GH18 family is widely distributed in all kingdoms, including viruses, bacteria, plants, fungi and animals [3]. Chitinases from the GH19 family are found mainly in plants [11], but they have also been described in bacteria, viruses and nematodes [12]–[14]. All fungal chitinases described thus far belong to the GH18 family, and this family is derived from an ancient gene family widely expressed in Archaea, Bacteria and Eukarya [15]. Besides chitinases, the GH18 family also contains endo-β-N-acetylglucosaminidases (EC 3.2.1.96), known as ENGases, which play a role in deglycosylation [5].

The number of chitinase genes displays a wide variation in fungal genomes, from a single gene in *Schizosaccharomyces pombe* up to 36 genes in *Trichoderma virens* [16]. Eighteen chitinase sequences were found in the *Trichoderma reesei (Hypocrea jecorina)* genome, and the chitinase domain composition was used to classify them into subgroups A, B and C [17]. Later, an expanded chitinase classification included bacteria, archaea, viruses, fungi, plants and animals [18]. Some of the different domains found in fungal chitinases to date are (i) the GH18 domain, containing the DxxDxDxE and S/AxGG conserved regions, (ii) the carbohydrate-binding module CBM18 ( = ChBD, chitin-binding domain), (iii) the CBM1 ( = CBD, cellulose/chitin-binding domain), (iv) the CBM50 ( = LysM domain), and (v) a serine/threonine-rich region [4]. Recently, the entire genomes of different species from the genus *Metarhizium* were sequenced (*Metarhizium robertsii*, *Metarhizium acridum* and the teleomorphic state of *Cordyceps militaris*), and they were found to contain numerous chitinase genes [19], [20]. These highly infective entomopathogenic fungi have the capability to differentiate into diverse cellular types (mycelia, conidia, appressoria and blastospores) during the host-infection cycle [21]. The fungal cell wall frequently undergoes remodeling as the cell expands and develops. Chitinases and chitin synthases act in remodeling the fungal cell wall in order to switch among these developmental cell types [22]. Beyond cell type modifications and cell wall remodeling, the potential fungal chitinase functions include exogenous chitin degradation as a nutrient source acquisition strategy and competition and defense against fungi and arthropods [4], [23]. Functional analyses from fungal chitinases are expanding the roles assigned to the members of this multigene family. The most studied chitinase genes belong to subgroup A (sgA) and B (sgB), followed by chitinase genes from subgroup C (sgC), which are highly represented among *Trichoderma* species [24]. As reviewed by Hartl et al. [23], sgA is believed to contain enzymes involved in fungal growth and autolysis, sgB proteins seem to participate in nutritional and virulence functions, and sgC may have several roles in self and non-self chitin degradation.

In *M. anisopliae*, only two chitinase genes from sgB (*chi2* and *chi3*) were individually deleted, and both deleted strains showed diminished virulence in insect bioassays [25], [26]. SgA proteins from *Trichoderma* and *Aspergillus* species were suggested to be involved in autolysis [27]–[29]. Members from sgC chitinases were initially studied in *Trichoderma* spp., where the transcript profiles showed multiple induction conditions (during mycoparasitism, during hyphal network formation and by chitin) [24]. Recently, this subgroup was also analyzed in *A. nidulans*, and the sgC-II chitinases were suggested to be involved in fungal-fungal interactions [30]. Although several chitinases from filamentous fungi and yeasts have been isolated and characterized, the exact physiological functions of these enzymes remain to be determined. The assumption that the genes from the same subgroup present a corresponding functionalization may not reflect the biological purpose of the corresponding proteins. The alternative hypothesis that substrate accessibility is the principal factor determining chitinase activity also remains to be established [16]. Additionally, not only must mutant strains and chitinase expression levels be evaluated, but it is also necessary to clarify the function of these enzymes at the biochemical level because the evidence that they are all active is still lacking.

Here, we described a survey of the putative chitinase coding genes from one of the best-characterized fungal entomopathogens, *M. anisopliae*. On a genomic scale, we identified 21 new/unknown GH18 genes in addition to the three that were previously described, *chit1*, *chi2* and *chi3* [26], [31]–[33]. An *in silico* analysis of those enzymes was performed, and they were subsequently classified as A, B, C as well as two novel subgroups, D (sgD) and E (sgE). The gene structures of the identified chitinases (sg A, B, C and D) and ENGases (sgE), including their conserved domain organizations, intron contents, and evolutionary histories were evaluated. The predicted GH18 domain-containing genes (hereafter called GH18 genes) were validated through transcript detection under different growth conditions. To gain information regarding the possible roles of these gene products in different cell types, the relative transcript levels from 23 GH18 genes were evaluated by quantitative RT-PCR (qPCR) under different conditions. These results open up new possibilities for studying the participation of chitinases in fungal biology and indicate the most relevant candidates for further functional analyses.

## Materials and Methods

### Fungal Strains and Culture Conditions

The *M. anisopliae* E6 strain used in all analyses was isolated from the insect *Deois flavopicta* in Brazil [34]. *M. anisopliae* E6 was cultured under eight different growth conditions prior to RNA extraction. Conidia were harvested from cultures in agar plates and glass wool filtered to remove mycelium. *M. anisopliae* conidial suspensions (1×10^6^ conidia.mL^−1^) were inoculated into different culture media. Cove’s Complete medium (MCc) composition was (w/v): 1% glucose, 0.6% NaNO_3_, 0.15% casein hydrolisate, 0.05% yeast extract, 0.2% peptone, pH 7,0 plus 2% (v/v) Salts Solution (2.6% KCl, 2.6% MgSO_4_•7H_2_O and 7.6% KH_2_PO_4_ (w/v)) and 0,04% (v/v) Trace Elements Solution (4 mg % Na_2_Ba_4_O_7_•7H_2_O, 40 mg %CuSO_4_•5H_2_O, 1 mg % FeSO_4_, 80 mg % Na_2_MNO_4_•7H_2_O, 80 mg % MnSO_4_•7H_2_O and 80 mg % ZnSO_4_•7H_2_O (w/v)). Minimum medium was composed of 0.6% NaNO_3_ (w/v) plus carbohydrate source (0.25% N-acetylglucosamine (GlcNAc) (w/v) or 1% crystalline chitin from crab shells) with Salts and Trace Elements Solutions. Cultures were maintained on a shaker (180 rpm) for 72 h at 28°C. Mycelium from autolysis induction medium (1% glucose (w/v) and 0.6% NaNO_3_ (w/v) for 9 days) was also collected. For harvesting, the mycelia were abundantly washed with sterile dH_2_O, filtered through Miracloth and frozen in liquid nitrogen for total RNA extraction. In addition to mycelial samples, total RNA from different cell types was extracted from conidia, blastospores and appressoria, and from 24 h fungal growth on *Rhipicephalus microplus* cuticles as an infection model. Appressoria induction was performed with 5×10^5^ conidia.mL^−1^ inoculated in 0.004% yeast extract solution on 500 glass coverslips for 16 hours at 28°C. For blastospore production, 5×10^4^ conidia.mL^−1^ were cultured in ADAMEK medium (3% Corn Steep Solids, 4% glucose and 3% yeast extract (w/v)) in a shaking platform for 64 hours at 28°C. Appressoria and blastospore induction was confirmed by observing randomly picked coverslips under a microscope (Figure S1). RNA extraction for all eight conditions was performed in three replicates. All reagents used were supplied from Sigma-Aldrich.

### Chitinase Gene Biomining in the *M. anisopliae* Genome

*M. anisopliae* E6 pyrosequencing was performed at Laboratório Nacional de Computação Científica (LNCC, RJ, Brazil), resulting in 23× genome coverage (Staats *et al*., to be published elsewhere) (accession number PRJNA245858). The contigs resulting from the genome draft assembly were used to identify the GH18 genes with selected yeast and filamentous fungal chitinase sequences as queried in the tBLASTn program. The fungal species and their corresponding strains used in this search are listed in Table S1. All sequences were extracted from the BROAD Institute and NCBI databases. First, we used 20 *T. reesei* amino acid sequences [17] to identify chitinases belonging to these microorganisms. Subsequently, each identified chitinase was used in the search for positive similarity in *M. anisopliae* contigs employing the tBLASTn algorithm with the BioEdit software [35]. The positively identified chitinases were screened for the presence of the GH18 family domain. The same screening methodology was applied using 91 reviewed fungal chitinase sequences extracted from the SwissProt database as well as the DxxDxDxE conserved domain.

To verify the presence of potential GH19 family proteins in the *M. anisopliae* genome, *Pringlea antiscorbutica* and *Arabidopsis thaliana* amino acid sequences (accession nos. AAP94636.1 and NP_188317.2, respectively) were also used in the previously described screening process.

### GH18 Genes Sequence Analyses

The *in silico* identified GH18 genes were confirmed by comparing the predicted sequences obtained from *M. anisopliae* contigs to public databases. To categorize a predicted protein sequence as a chitinase or ENGase, it should exhibit the GH18 family domain with the two frequently detected regions S/AxGG and DxxDxDxE. The sequence may also exhibit other characteristic domains such as the carbohydrate-binding modules CBM18, CBM1 and CBM50 typically found on fungal chitinases. These CBMs were traditionally defined as chitin binding domain (ChBD, IPR001002), cellulose/chitin binding domain (CBD, IPR000254) or LysM domain (IPR018392) [4], [36]. All domains were analyzed using the InterProScan [37], dbCAN [38] and CDD databases (Conserved Domain database) [39] provided by NCBI. The theoretical signal peptide cleavage site and the GPI anchor prediction for each protein were evaluated using the SignalP 4.1 server [40] and the big-PI Fungal Predictor software [41]. Theoretical isoelectric point and molecular mass values were obtained from the Compute p*I*/Mw software [42]. The ScanProsite server was used to detect the number of N-glycosylation sites. The number and positions of introns were predicted for each new chitinase by comparison with database chitinase sequences using BLASTx. The flanking regions were screened for the presence of canonical 5′ and 3′ splice sites. The positional insertion of these sequences among chitinases was also analyzed.

### GH18 Proteins Phylogenetic Analyses

The *M. anisopliae* and *T. reesei* chitinase and ENGase amino acid sequences corresponding to mature proteins (excluding the signal peptide) or to the GH18 domain were aligned by the ClustalW software [43] using the BLOSUM matrix and additional default parameters. Phylogenetic trees were constructed in MEGA 6 [44], using the Neighbor-Joining algorithm [45], the pairwise deletion for the gap treatment and the p distance, Poisson and JTT matrices. The bootstrap test of phylogeny was performed with 1,000 repetitions. The OrthoMCL v2.0.8 [46] software was used with default parameters to identify the orthologs and paralogs of chitinases among *M. anisopliae* E6 and fifteen other predicted fungal proteomes: *Aspergillus fumigatus* Af293, *Aspergillus nidulans* FGSC A4, *Aspergillus niger* CBS 513.88, *Beauveria bassiana* ARSEF 2860, *Cordyceps militaris* CM01, *Fusarium graminearum* PH-1, *Fusarium oxysporum* f. sp. cubense race 1, *M. robertsii* ARSEF 23, *M. acridum* CQMa 102, *Magnaporthe oryzae* 70-15, *Neurospora crassa* OR74A, *Nectria haematococca* mpVI 77-13-4, *Trichoderma atroviride* IMI 206040, *Trichoderma reesei* QM6a and *Trichoderma virens* Gv29-8. To infer the phylogenetic relationship among the three *Metarhizium* strains (*M. anisopliae* E6, *M. robertsii* ARSEF23 and *M. acridum* CQMa102) and *C. militaris*, a phylogenetic tree was constructed using the *tef-1-*α gene (Figure S2).

### RNA Sample Preparation and Transcript Analyses

Total RNA from *M. anisopliae* cells obtained under different growth conditions was extracted by standard procedures using Trizol Reagent (Life Technologies, Grand Island, NY, USA) on powder samples grinded on mortar and pestle with liquid nitrogen. Residual DNA was submitted to DNase treatment (Thermo Scientific, MA, USA) and, then, to an RNeasy Cleanup column (Qiagen, Hilden, Germany). One µg of total RNA, quantified on a Qubit fluorometer (Life Technologies, Grand Island, NY, USA), was used for cDNA synthesis using MMLV-RT enzyme (Life Technologies, Grand Island, NY, USA). The procedures were performed according to the manufacturers’ instructions, and all RNA samples were stored at –80°C. To validate chitinase gene predictions, the transcript detection was accomplished by RT-PCR. RT-PCR primers for each chitinase gene were designed for the flanking intron sequences to observe differential band patterns when compared to genomic DNA. Considering the conservation of chitinase genes, the design of oligonucleotides for PCR involved searching specific regions for each gene (Table S2).

### Quantitative Real-Time PCR (qPCR) Experiments

All samples from the different conditions were analyzed in three biological replicates. NTC (no template control) and NRTC (no reverse transcriptase control) negative controls were included in each experiment. qPCR primers were designed using the Primer Premiere 6 software (PREMIER Biosoft, Palo Alto, CA, USA) and by selecting the “Avoid Cross Homology” tool option to obtain specificity and prevent designing primers in homologous sequence regions among members of the chitinase family (Table S2). γ-actin was used as the reference gene transcript (accession no. MANI05119). The transcript relative quantification from each gene was performed using the Platinum SYBR Green qPCR SuperMix-UDG kit (Life Technologies, Grand Island, NY, USA) with StepOne equipment (Applied Biosystems, Foster City, CA, USA) and the StepOne 2.2 software (Applied Biosystems, Foster City, CA, USA). The specificity from the synthesized products and the absence of primer dimers were visualized in melting curve analysis for each reaction. The amplification efficiency for each individual sample from MCc condition was calculated using LinRegPCR software application [47] and the mean efficiency values for each primer were added to Table S2. The same efficiency value was used for the quantification analysis. Transcript expression was calculated by analyzing Cq (quantification cycle) values and using 2^−ΔCt^ and 2^−ΔΔCt^ methods [48]. The results were processed in the GraphPad Prism (La Jolla, CA, USA) and GenePattern software [49] for graphics and statistical data acquisition, respectively. Statistical data were obtained by performing a one-way analysis of variance (ANOVA) test followed by Tukey’s multiple comparisons test (P<0.05) to compare the 2^−ΔCt^ and 2^−ΔΔCt^ values of the eight experimental groups and to determine significant differences.

## Results

### The 24 *M. anisopliae* GH18 Genes can be categorized into Five Subgroups

The screening of *M. anisopliae* contigs using the 20 *T. reesei* chitinase and ENGase protein sequences as queries allowed the identification of 26 positive hits. All other fungal sequences used as queries (including *M. robertsii*, *M. acridum* and *C. militaris* sequences) resulted in alignments with the same 26 previously detected contigs. Several other sequences showed diverse similarity levels with the query sequences. Nevertheless, only these 26 identified sequences included the GH18 domain, which is considered indispensable for chitinase categorization. By analyzing these sequences, 24 putative chitinase ORFs were identified, including the three previously isolated *M. anisopliae* chitinase genes (*chit1*, *chi2* and *chi3*) [31]–[33]. The GH18 domain was identified in all predicted genes using InterProScan, CDD and dbCAN. The two remaining sequences, although possessing the GH18 domain and other chitinase characteristics, presented several stop codons interrupting important coding regions and were therefore considered pseudogenes.

Based on the subgroup chitinase classification by Seidl et al. [17], 20 out of the 24 predicted GH18 proteins could be attributed to subgroups A, B and C. The *M. anisopliae* chitinase categorization was performed by domain detection and alignment analysis against *T. reesei* classified chitinases. A phylogenetic tree of the 24 *M. anisopliae* and 20 *T. reesei* chitinases and ENGases was constructed (Figure S3). Most of the *M. anisopliae* chitinases clustered with corresponding *T. reesei* chitinase subgroups A, B and C. Of the 24 predicted chitinases, nine were assigned as subgroup A, seven were assigned as subgroup B, and four were assigned as subgroup C. Moreover, four remaining GH18 proteins were grouped in a separated branch and one of them presented higher similarity with *T. reesei* chi18-15, which was not included in subgroup A, B or C and was not added to the phylogenetic trees constructed by Seidl et al. [17]. Later, although chi18-15 was not assigned as part of a specific fungal subgroup, it was shown to be near subgroups B-V and B-I by Karlsson & Stenlid [50]. The other three proteins in this separated branch grouped with GH18 enzymes characterized as ENGases [51]. In *M. anisopliae* GH18 proteins classification, the protein ortholog to *T. reesei* Chi18-15 was assigned to a new subgroup D following the existing classification, and the three proteins similar to ENGases were assigned to a new subgroup E (Figure S3). Two major branches can be observed in Figure S3, one including subgroups A and C and another clustering subgroups B, D and E. *M. anisopliae* putative chitinases were named ChiMaAs (ChiMaA1 to ChiMaA9), ChiMaBs (ChiMaB1 to ChiMaB7), ChiMaCs (ChiMaC1 to ChiMaC4) and ChiMaD (ChiMaD1); and *chima* was used for the gene nomenclature. The three putative ENGases were named MaEng18A, MaEng18B and MaEng18C according to their *Trichoderma* orthologs. A tBLASTn search for potential GH19 chitinase domains using two amino acid sequences from the GH19 family as queries did not render any hits within the *M. anisopliae* contigs, suggesting the absence of this plant-chitinase family.

**Figure 1:**
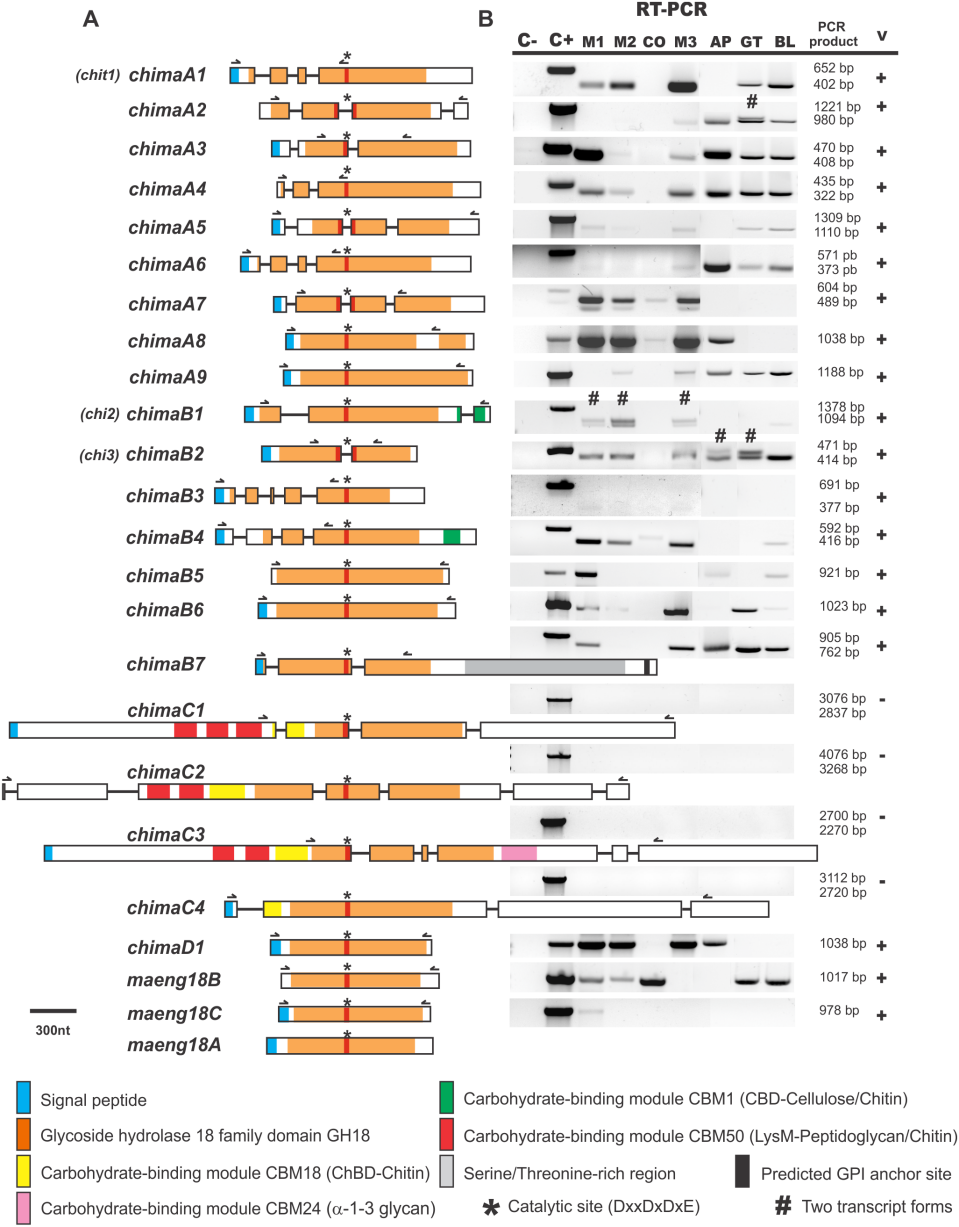
Modular domain structure and transcripts from *M. anisopliae* GH18 genes. **A)** GH18 genes exhibit characteristic conserved domains with different compositions. Coding exonic sequences are depicted as boxes (color codes are indicated) and introns as thin lines. Domains were identified using Conserved Database Domain (at NCBI), dbCAN and InterProScan. Signal peptide sequences were predicted using SignalP 4.1. Blank protein regions indicate the absence of characterized domains. Horizontal semi-arrows indicate oligonucleotide positions and directions used for transcript validation. **B)** The validation of GH18 genes was achieved by transcript detection (+ or – on V column) with RNA extracted from seven different culture conditions in RT-PCR reactions using specific primers shown by the semi-arrows on panel A. The length of PCR products is displayed as bp and compared to C+ (genomic DNA as template) and C− (no template added). RNA extracted from: **M1 -** **mycelium** grown in complete medium (MCc); **M2** - mycelium grown in **induction GlcNAc** 0.25% medium; **CO -** **conidia** suspension; **M3 -** mycelium under **autolysis**; **AP -** from cultures under **appressorium** induction; **GT -** from cultures under **germ tube** formation induction; **BL -** from cultures in **blastospore** induction medium. #indicates alternative transcript forms detected.

The structural domains frequently found in chitinases – GH18, CBM18, CBM1 and CBM50- were also detected in *M. anisopliae* predicted proteins by using the Conserved Database Domain (at the NCBI server), dbCAN database and InterProScan software. The GH18 domain, which is conserved in these enzymes, was found in all predicted chitinase sequences as well as in ENGase sequences (Figure 1). This domain contains the two conserved regions, DxxDxDxE and S/AxGG, corresponding to the catalytic and substrate binding regions, respectively (Figure S4). Amino acid alignment showed that all chitinases have a glutamate (E) residue in the conserved region DxxDxDxE, which has been shown to be essential for catalytic activity [52]. The absence of this specific residue is characteristic of chitolectins, chitin-binding proteins that are not able to hydrolyze the glycoside bond. The three aspartate residues (D) were also highly conserved, except for ChiMaB1 and MaEng18A (1^st^ aspartate residue) and ChiMaA2 (3^rd^ aspartate residue). The substrate-binding region S/AxGG showed a serine (S) or an alanine (A) for 21 out of the 24 GH18 proteins, except for sgE proteins, MaEng18A, MaEng18B and MaEng18C, which exhibit a methionine (M) residue (Figure S4). GH18 was the only domain exhibited by sgA and sgD chitinases and by sgE ENGases. A CBM1 domain lying at the protein C-terminal end was detected on only two (ChiMaB1 and ChiMaB4) out of seven sgB chitinases (Table 1). Seidl [4] observed that *B. cinerea*, *S. sclerotiorum* and *Trichoderma* spp. have several sgB chitinases with a CBM1 domain. For instance, four out of five *T. reesei* sgB chitinases presented the CBM1 domain, but *Aspergillus* spp. do not spams as many CBM1 domains as *T. reesei* sgB chitinases does. The same is observed for *M. anisopliae* sgB chitinases. One of the sgB chitinases (ChiMaB7) presented a large (approximately 326 amino acids) serine and threonine-rich domain. This ser/thr-rich domain was also found in other fungal chitinases and may take part in protein folding [4] or may be involved in the membrane localization (GPI anchor) of this protein. The high molecular mass chitinases from sgC (>120 kDa) exhibited multiple CBM50 domains. Three out of four sgC chitinases have one to three CBM50 domains in their sequences, which are thought to bind to peptidoglycan-like and chitin oligosaccharides [53]. Another carbohydrate-binding domain detected in sgC chitinases was the CBM18, containing eight cysteine residues, which may form disulfide bonds. Those extra carbohydrate-binding domains are thought to enhance substrate-enzyme coupling, but the exact contribution to the enzyme activity/function is still unknown. As analyzed by Gruber et al. [24], sgC chitinases have two types of domain organization. In *M. anisopliae* sgC chitinases, ChiMaC1, C2 and C3 have the first type of architecture, with the GH18 domain localized in the middle of the sequence, and ChiMaC4 has the second type, characterized by an N-terminal location of the GH18 domain and no CBM50 domain. One CBM24 domain, never before reported in GH18 chitinase sequences, was detected using dbCAN annotation tool. The CBM24 seems to bind α-1-3 glucans and it is present in α-1-3 glucanases [54]. The CBM24 domain was found at the C-terminal region of ChiMaC3.

**Table 1:** Properties of the 24 *M. anisopliae* glycoside hydrolase family 18 genes (GH18 genes).

Identification	Sg	SignalP	CBMs	GPI or NCS	Mature proteintheoretical kDa	Accession number
**ChiMaA1 (** ***chit1*** )*	A	+	−	−	44.12 **(42)**	MANI7345
ChiMaA2	A	−	−	+NCS	38.60	MANI3521
ChiMaA3	A	+	−	−	42.62	MANI22967
ChiMaA4	A	−	−	−	44.18	MANI12760
ChiMaA5	A	+	−	−	38.78	MANI5739
ChiMaA6	A	+	−	−	43.49	MANI29841
ChiMaA7	A	+	−	−	39.36	MANI730
ChiMaA8	A	+	−	−	41.67	MANI21851
ChiMaA9	A	+	−	−	41.20	MANI6991
**ChiMaB1 (** ***chi2*** )*	B	+	−	−	41.96 **(42)**	MANI2801
**ChiMaB2 (** ***chi3*** )*	B	+	CBM1	−	32.16 **(30/32.4)**	MANI4755
ChiMaB3	B	+	−	−	32.71	MANI26679
ChiMaB4	B	+	CBM1	−	42.95	MANI4417
ChiMaB5	B	−	−	+NCS	33.02	MANI21602
ChiMaB6	B	+	−	−	33.30	MANI18482
ChiMaB7	B	+	−	+GPI	80.63	MANI12994
ChiMaC1	C	+	CBM18 and 3 CBM50	−	148.23	MANI23684
ChiMaC2	C	−	CBM18 and 2 CBM50	−	127.58	MANI19486
ChiMaC3	C	+	CBM18, 2 CBM50, CBM24	−	168.76	MANI10050
ChiMaC4	C	+	CBM18	−	125.68	MANI30406
ChiMaD1	D	+	−	−	33.81	MANI18860
MaEng18A	E	+	−	−	38.57	MANI23769
MaEng18B	E	−	−	+NCS	37.58	MANI9126
MaEng18C	E	+	−	−	34.33	MANI30302

*M. anisopliae* chitinases and ENGases identification, subgroup classification, molecular mass and accession numbers are given. The presence of predicted signals and carbohydrate-binding motifs (CBM) detected on the 24 GH18 proteins are marked as positive (+). These include presence of signal peptides (SignalP), GPI-anchors (GPI) and regions recognized by non-classically secretion pathways (NCS). Proteins marked with *were previously studied in *M. anisopliae* E6, as shown in references 25, 26, 31–33. At mature protein kDa column, the experimentally observed molecular mass is indicated between parentheses.

### *M. anisopliae* GH18 Protein Properties and Predicted Subcellular Localization

The predicted *M. anisopliae* E6 GH18 ORFs were characterized by *in silico* prediction tools, based on the sequence length, molecular mass and isoelectric point. The protein lengths ranged from 306 to 1,556 amino acids and 33.02 to 168.76 kDa. Fifteen out of 24 predicted proteins presented pI<6.0, four presented pI>7.0 and five presented pI at approximately 6.0 (Table S3). These data are in agreement with the vast diversity of lengths observed for chitinases: 27 to 190 kDa and pI between 3.0 and 8.0, as previously reviewed [3]. The chitinases from sgC are larger than the proteins from the other subgroups. Predicted localization signals and post-translational modification of the 24 *M. anisopliae* GH18 proteins were also evaluated. The localization analysis included the presence of signal peptides, transmembrane regions, GPI-anchors and amino acid regions recognized by non-classical secretion pathways. Nineteen out of 24 predicted protein sequences contained a theoretical signal peptide and corresponding cleavage sites (Figure 1, Table 1). The signal peptide sequences presented a hydrophobic central domain and an alanine residue at position −1, as is characteristic of eukaryotes [55], [56]. At least one member of each subgroup (except sgD) did not present signal peptide sequences (ChiMaA2, A4, ChiMaB5, ChiMaC2 and MaEng18B). The presence of signals for GPI anchors was evaluated using the Big-PI Fungal Predictor software. Only one chitinase presented a potential GPI-modification site (ChiMaB7) (Table 1). Additionally, the subcellular prediction analyzed by WolfPSORT showed that ChiMaA2, which has no evidence of a signal peptide, might be located at the mitochondrial matrix, similarly to its ortholog chi18-3 from *T. reesei* [17]. Finally, post-transcriptional modifications were predicted for *M. anisopliae* chitinases, and the predicted proteins showed high levels of glycosylation (Table S3).

### Intron Analyses in Chitinase Genes

Intron prediction considering the number, length and position was performed using BLASTx. All chitinase genes from sgC and most genes from subgroups A and B contain introns. However, sgD and sgE genes are not interrupted by intron sequences (Figure 1), as is the case for the ChiMaA8, A9, B5 and B6 sequences. Subgroups A and B presented averages of 2.7 and 2.4 introns per gene, and sgC showed a higher intron content (4.25 introns/gene), which agrees with the increased length of the genes exhibited by this subgroup. Intron length values were not stable for all sequences. Intron sequences were longer in sgC genes (ca. 100 nt) than in subgroups A and B (68 and 78 nt, respectively). All introns exhibited the canonical 5′ (5′ GU) and 3′ (AG 3′) splice sites on flanking regions. The intron positions are depicted in Figure 1. Analysis of the intron positional insertion was performed by comparison among chitinase sequences. It was observed that, for the majority of the genes, intron positions were not conserved among subgroups, although a noticeable pattern of intron insertion near the region containing the catalytic conserved active site was identified. This catalytic site appears to be interrupted by an intron in four genes (A2, A5, A7 and B2) and flanked by an intron in another four genes (A3, B7, C1 and C3). Considering other insertion positions, the majority of introns is placed before this catalytic site at the N-terminal region. Finally, all introns contained in ChiMaB3 and B4 showed very similar intron positions, while ChiMaA5 and A7 exhibited identical intron positions (Figure S5).

### Phylogenetic Analyses grouped GH18 Protein Orthologs into Five Distinctive Subgroups

**Figure 2:**
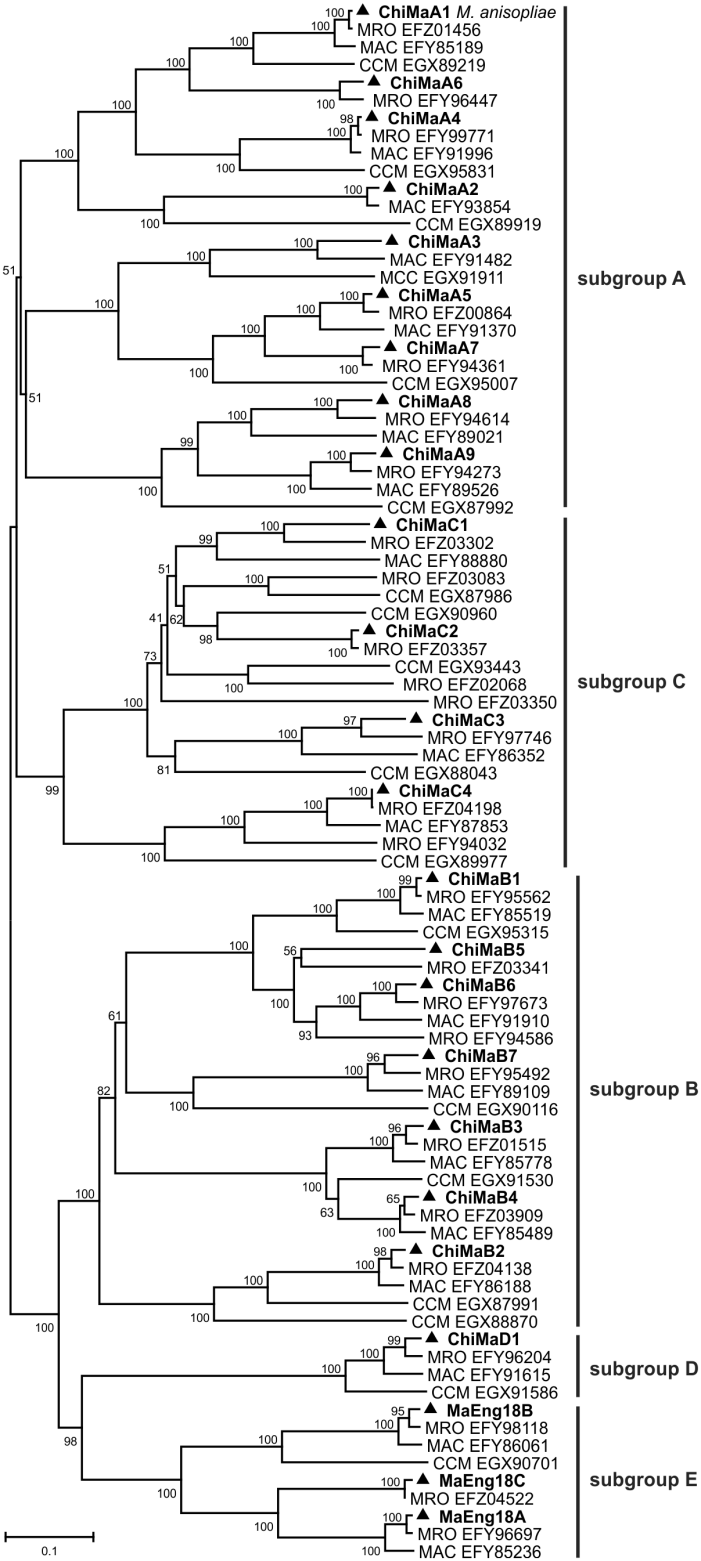
Phylogenetic relationships among GH18 domain-containing proteins and subgroup divisions in three *Metarhizium* strains and *Cordyceps militaris*. Amino acid sequences corresponding to GH18 domains from *M. anisopliae* chitinases and ENGases (marked as ▴), *M. acridum* (MAC), *M. robertsii* (MAA) and *C. militaris* (CCM) were obtained from the NCBI databases. The Neighbor-Joining (1000 bootstraps) phylogenetic tree was constructed using Mega 6 after ClustalW alignment. The scale bar indicates the genetic distance, which is proportional to the number of amino acid substitutions.

All alignments were obtained using only the GH18 domains because the additional regions of the genes showed high levels of variability. Similar procedures were applied in previous studies [50]. *M. robertsii, M. acridum* and *C. militaris* GH18 protein sequences were compared to *M. anisopliae* predicted proteins. The resulting phylogenetic tree displayed the same subgroup arrangement that was identified when comparing *M. anisopliae* to *T. reesei* chitinases (Figure S3). Two main clades were obtained, one including the A and C subgroups and the other formed by the B, D and E subgroups (Figure 2). The number of GH18 proteins detected in the *M. anisopliae* genome was intermediate (24 proteins) when compared to *M. robertsii* (28 proteins), *M. acridum* (19 proteins) and *C. militaris* (19). The chitinase sequences from *M. robertsii* and *M. acridum* were more closely related to *M. anisopliae* proteins than to *C. militaris* proteins. Considering these three anamorphic species, not all chitinase members were detected in the three species. Six out of nine sgA chitinases were present in the three species, while ChiMaA6 and A7 were absent in *M. acridum*, and ChiMaA3 was absent in *M. robertsii*. Six out of seven members from sgB are shared among species. Moreover, *M. acridum* lacks ChiMaB5, and *M. robertsii* has an exclusive member (accession no. EFY94586). *M. anisopliae* lacks four sgC proteins present in *M. robertsii*, whereas two of these sequences seem to be pseudogenes. The sgD protein was present in the four species (ChiMaD1); and *M. acridum* lacked sgE MaEng18C, and *C. militaris* lacked MaEng18A and MaEng18C.

**Figure 3:**
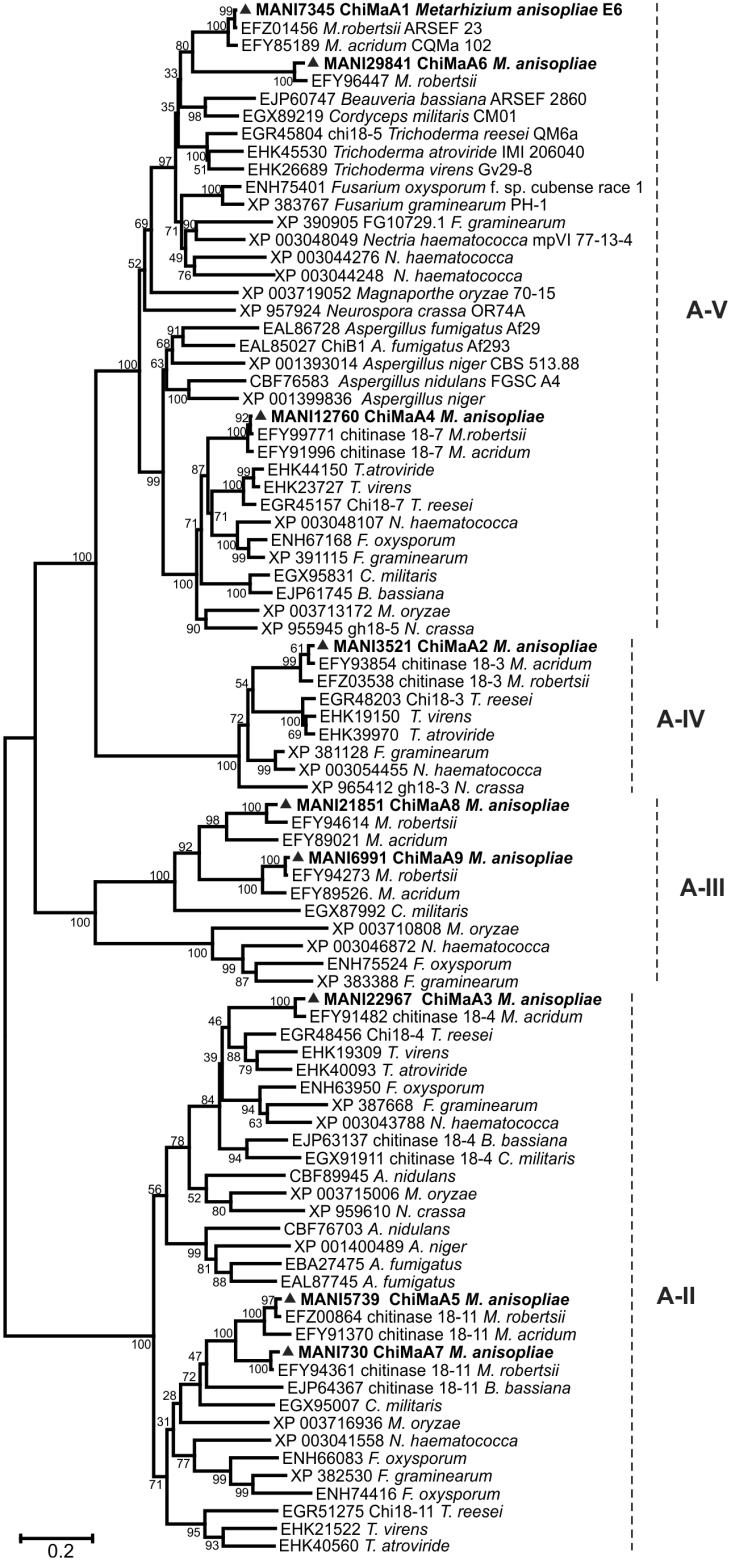
Phylogenetic tree of GH18 subgroup A chitinases. Chitinase amino acid sequences from *M. anisopliae* and other fungi were from the NCBI and BROAD Institute databases. The Neighbor-Joining (1000 bootstraps) phylogenetic tree was constructed using Mega 6 after alignment obtained from ClustalW. *M. anisopliae* chitinases are indicated as ▴ with their corresponding ID numbers.

The OrthoMCL analysis identified orthologs and paralogs among fungal genomes and allowed the construction of four phylogenetic trees based on subgroup classification. The sgA phylogenetic tree (Figure 3) displayed four branches equivalent with A-II, A-III, A-IV and A-V clades described by Seidl and Karlsson & Stenlid [17], [18]. Clades A-II and A-V present three *M. anisopliae* chitinases, clade A-III present two chitinases and clade A-IV only one chitinase. Clade A-V ChiMaA1 (CHIT42) orthologs are the most studied chitinases in several fungi. Clade A-II contains the only chitinase paralogs detected by OrthoMCL, ChiMaA5 and A7.

**Figure 4:**
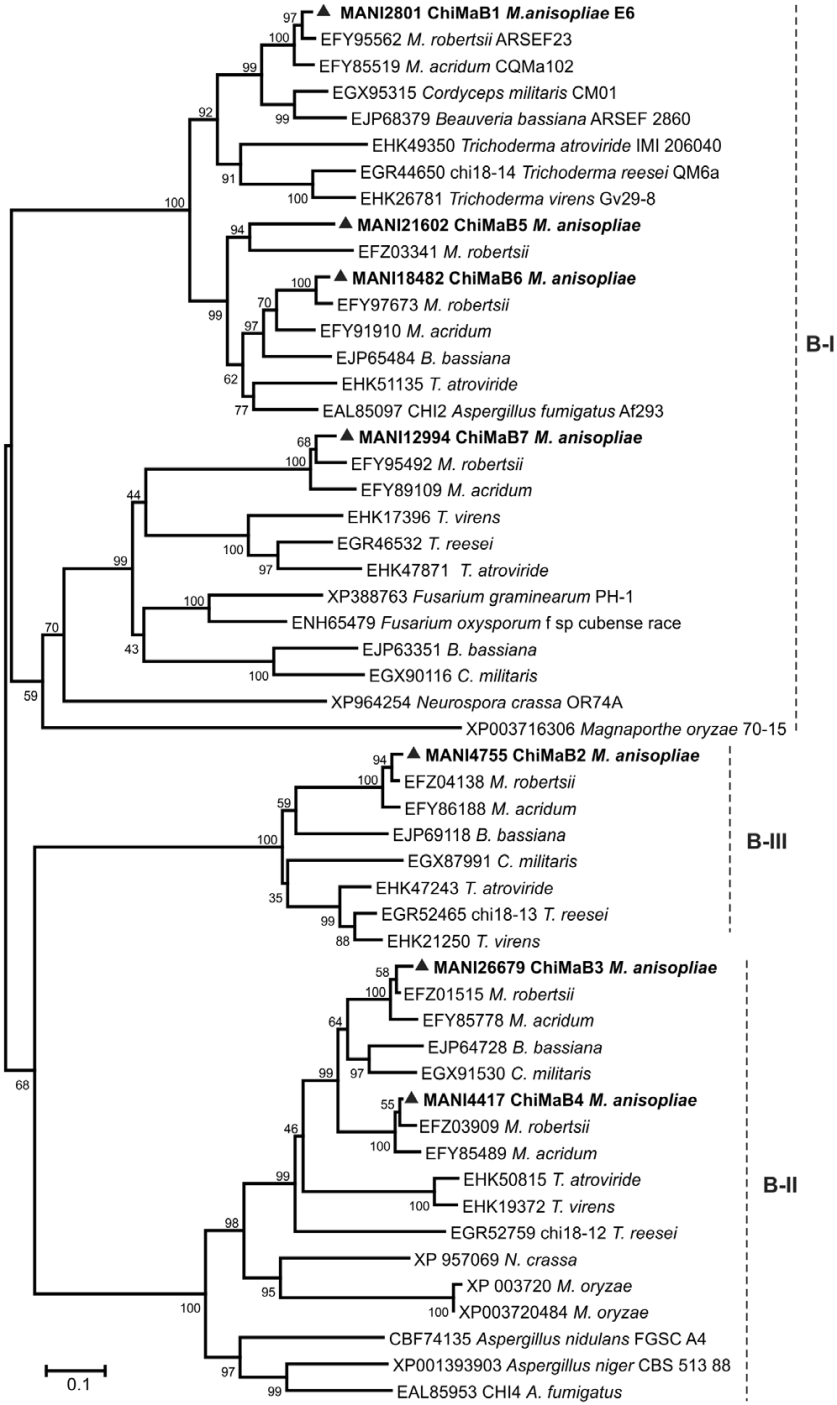
Phylogenetic tree of GH18 subgroup B chitinases. Chitinase amino acid sequences from *M. anisopliae* and other fungi were from the NCBI and BROAD Institute databases. The Neighbor-Joining (1000 bootstraps) phylogenetic tree was constructed using Mega 6 after alignment obtained from ClustalW. *M. anisopliae* chitinases are indicated as ▴ with their corresponding ID numbers.

Four well-supported major ramifications (clades B-I, B-II and B-III) can be observed in the sgB phylogenetic tree containing the seven *M. anisopliae* chitinase members (Figure 4). Clade B-I contains four sgB chitinases, the two closely related ChiMaB5 and B6, and also the Ser/Thr-rich chitinase ChiMaB7 and ChiMaB1. The two CBM1-containing chitinases are in this tree, ChiMaB1 and B4, in separate clades, B-I and B-II, respectively. The two previously studied chitinases from *M. anisopliae*, CHI2 and CHIT30 (ChiMaB1 and B2) were placed in this subgroup.

**Figure 5:**
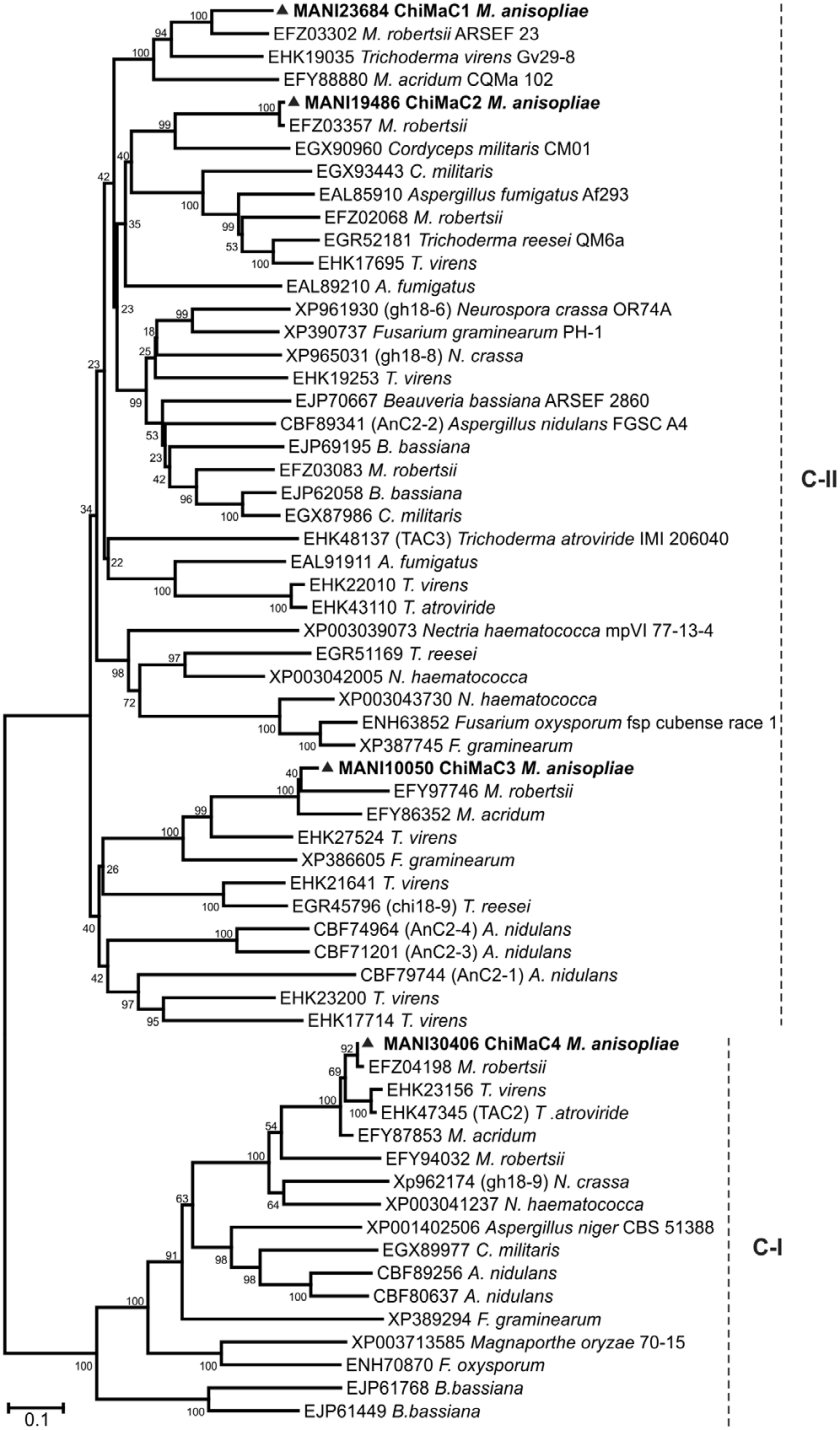
Phylogenetic tree of GH18 subgroup C chitinases. Chitinase amino acid sequences from *M. anisopliae* and other fungi corresponding to the GH18 domain were from the NCBI and BROAD Institute databases. The Neighbor-Joining (1000 bootstraps) phylogenetic tree was constructed using Mega 6 after alignment obtained from ClustalW. *M. anisopliae* chitinases are marked as ▴ with their corresponding ID numbers.

The SgC phylogenetic tree showed two major clades (Figure 5), the clade C-I with only one sgC member, ChiMaC4 and the clade C-II with the closely related ChiMaC1, C2 and C3. ChiMaC1, C2 and C3 have CBM50 domains, which are absent in the C4 chitinase, and the GH18 domain is located at the N-terminal protein region, whereas this domain has a central position in the sequences of the other three ChiMaCs. Although the division in clades C-I and C-II relates to the domain composition found in these proteins, only the GH18 domain was used to construct the tree, indicating that this domain *per se* also contains divergences. The sgC phylogenetic tree showed that *M. anisopliae* contains only four members, while *M. robertsii* has eight sgC chitinases. This subgroup has been shown to be highly overrepresented in *Trichoderma* spp., with 9 sgC chitinases in *T. atroviride* and 15 in *T. virens* [16]. Excluding *Trichoderma* species and *A. nidulans* [30], these high molecular mass chitinases remain poorly characterized.

**Figure 6:**
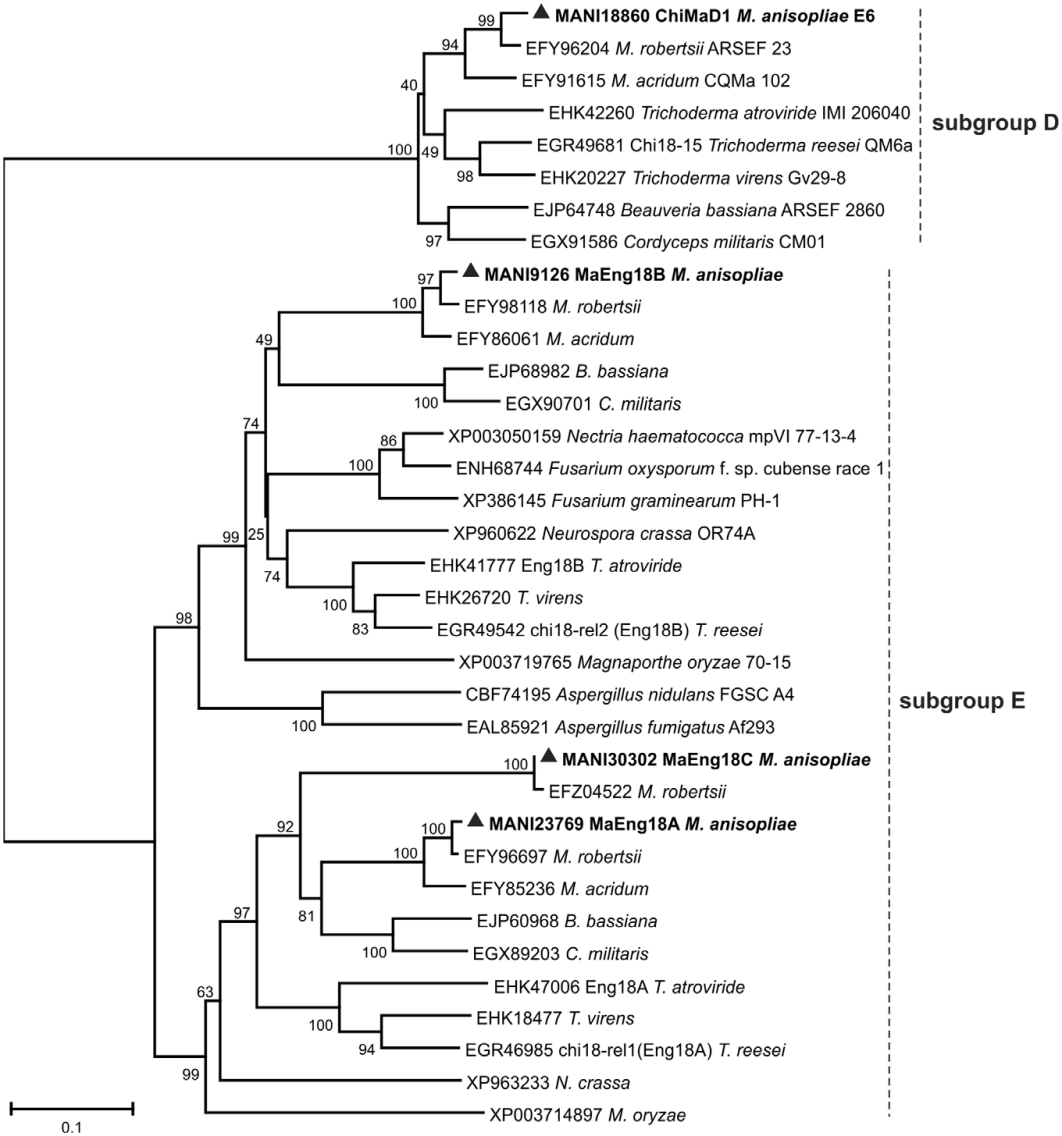
Phylogenetic tree of the GH18 subgroup D (chitinase) and subgroup E (ENGases). Amino acid sequences from *M. anisopliae* and other fungi were from the NCBI and BROAD Institute databases. The Neighbor-Joining (1000 bootstraps) phylogenetic tree was constructed using Mega 6 after alignment obtained from ClustalW. *M. anisopliae* chitinases/ENGases are marked as ▴ with their corresponding ID numbers.

A phylogenetic tree from the novel proposed subgroups D and E was also constructed and displayed three well-supported clades, D-I, E-I and E-II (Figure 6). Clade D-I contains ChiMaD1 and *T. reesei* chi18-15, clade E-I contains MaEng18B, and clade E-II contains MaEng18A and MaEng18C. Recently, orthologs from MaEng18A and MaEng18B (Eng18A and Eng18B from *T. reesei* and *T. atroviride*, as well as *Flammulina velutipes*) were proven to have a role in protein deglycosylation rather than chitinolytic activity [51], [57], [58]. GH18 proteins from these subgroups were not previously studied in *M. anisopliae*.

### Transcript Validation and Relative Expression Analyses Display Variable Transcript Profiles for Chitinase and ENGase Genes

**Figure 7:**
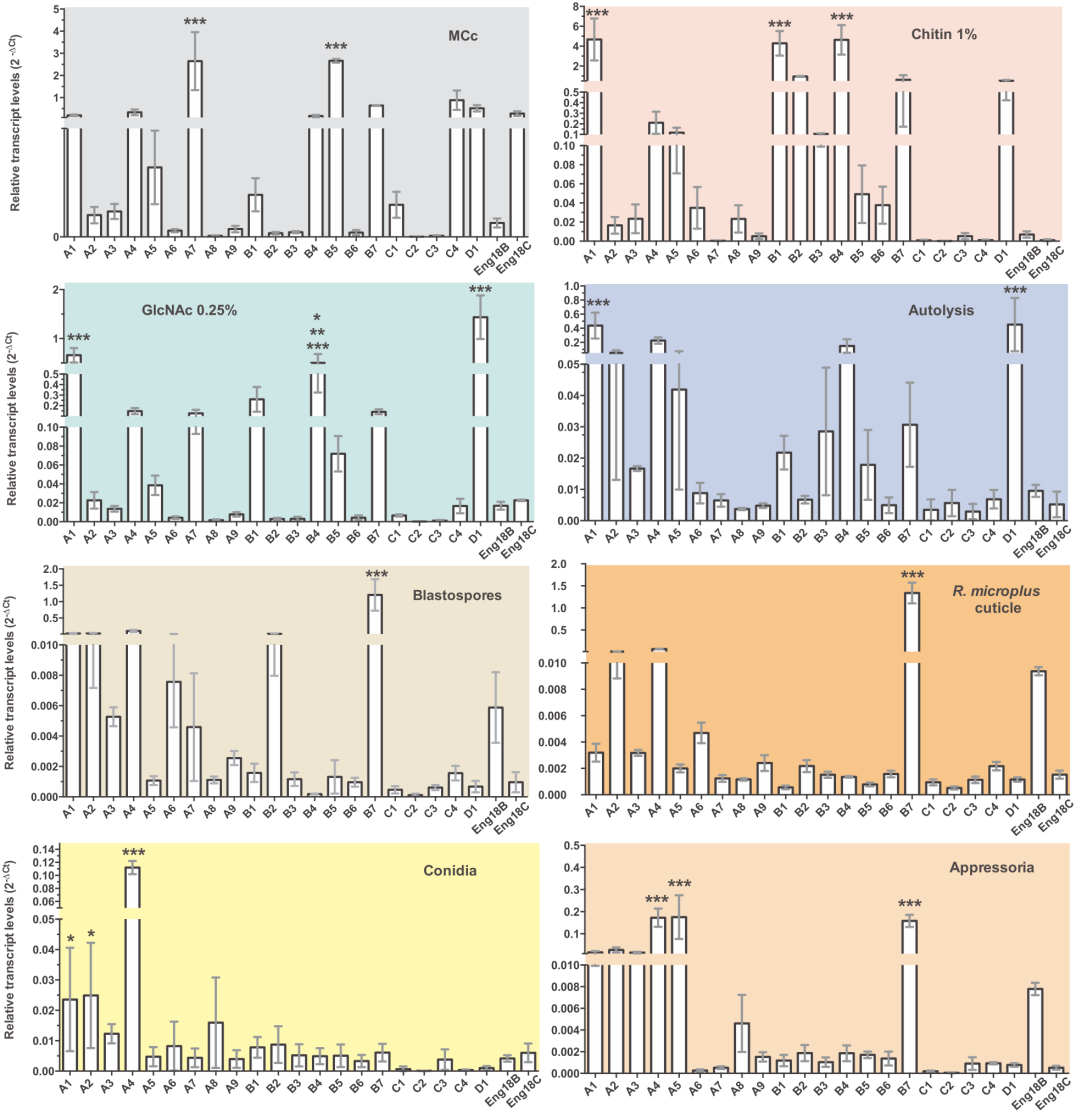
Relative chitinase and ENGase transcript profiles in *M. anisopliae*. Transcriptional profiles from 21 chitinase and 2 ENGase genes in eight different conditions using actin as the reference gene are displayed. The different cell types and culture conditions analyzed were mycelium grown on glucose, chitin 1%, GlcNAc 0.25% or autolysis conditions; and also induced blastospores, conidia, induced appressoria and 24 h fungal growth over tick cuticles. Data are shown as the mean ± SD from three experimental replicates of three biological replicates. *P<0.05, **P<0.01, ***P<0.001.

**Figure 8:**
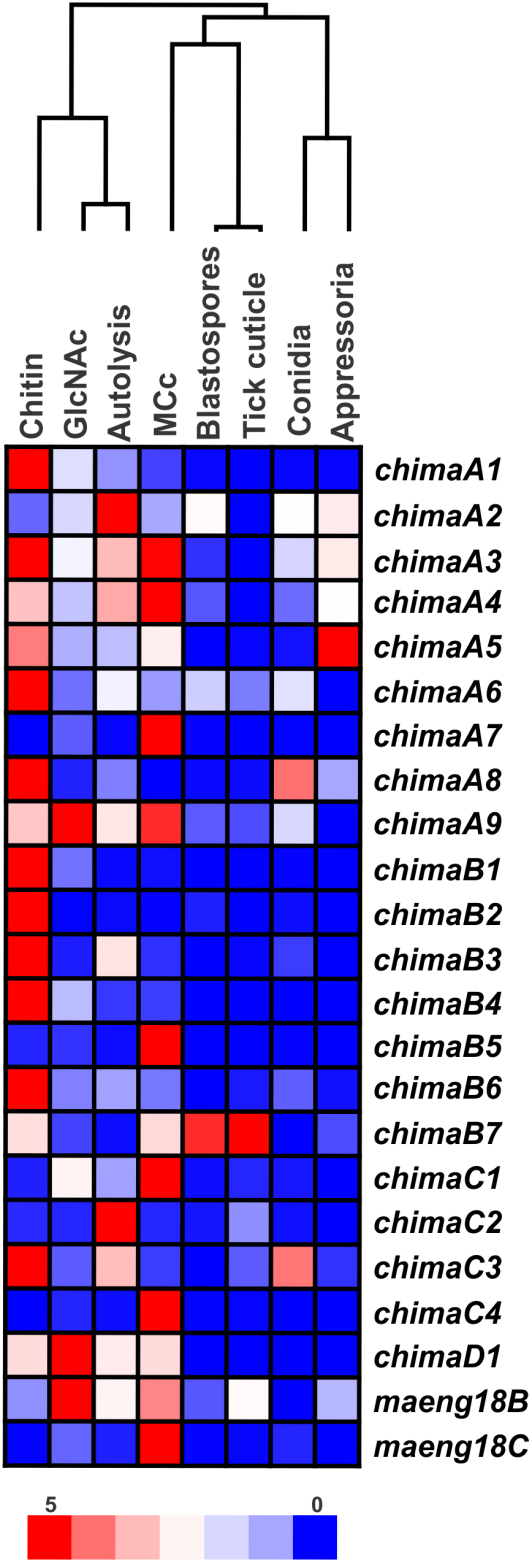
HeatMap and hierarchical clustering of the eight analyzed conditions by similar expression profiles. The HeatMap and hierarchical clustering were analyzed using GenePattern databases at BROAD Institute. The hierarchical clustering was used to group conditions (and not genes) by similar expression profiles. The highest transcript values are displayed as the reddest (hot), the lowest values are displayed as the bluest (cool), and intermediate values are a lighter color of either blue or red (2^−ΔCt^) for chitinase/ENGase in different conditions. Conditions are the same as in Figure 7.

Two methods (RT-PCR and qPCR) were used for transcription validation of *M. anisopliae* putative chitinases and ENGases. RNA was extracted from different cell types under different culture conditions: mycelium grown on glucose, chitin, GlcNAc or autolysis conditions; and induced blastospores, conidia, induced appressoria and 24 h fungal growth over tick cuticles. The last gene to be detected (*MaEng18A*) was absent from the transcriptional analyses, which were performed before its later detection. First, chitinase transcripts were detected by RT-PCR, and 19 out of 23 putative chitinase and ENGase genes produced detectable amplicons, including all members from subgroups A, B, D and E (Figure 1). However, SgC chitinase transcripts could be only detected by qPCR analysis. MCc and autolysis were the growth conditions where the higher number of GH18 transcripts was detected, with 17 and 16 GH18 transcripts, respectively. Conidia presented only four detectable chitinase transcript species (*chimaA7*, *A8*, *B4* and *MaEng18B*). As previously reported [59], the *chimaB1* gene (*chi2*) presented two forms of transcripts that could be detected in MCc, GlcNAc, autolysis and blastospore-inducing conditions. Moreover, two other chitinase genes (*chimaA2* and *chimaB*2) presented more than one potential transcript. These two different transcripts were detected in induced fungal germ tubes and appressoria. Secondly, transcript levels were also analyzed by qPCR. Single peak denaturation curves indicated primer specificity for each reaction. The 23 putative GH18 gene transcripts were detected in at least one of the different *M. anisopliae* cell types and culture conditions, validating the proposed annotation. The relative transcription levels of GH18 genes in comparison with γ-actin using 2^−ΔCt^ method were displayed in Figure 7 in order to highlight only the most pronounced transcripts and also to detect any pattern of regulation among the tested conditions (Figure 8). The most pronounced relative transcript levels from each subgroup were associated with the following genes: (i) *chimaA1* and *chimaA7*; (ii) *chimaB1*, *chimaB4*, *chimaB5* and *chimaB7*; (iii) *chimaC4*; and (iv) *chimaD1* and *MaEng18C* (Figure 7). *ChimaA1* showed high transcript levels with chitin and GlcNAc conditions and *chimaA7* showed high transcript level with MCc condition. Among sgB chitinases, *chiMaB7* showed significantly higher transcript levels than all other genes in the three conditions most related to infection (blastospores, tick cuticles and appressoria), and *chimaB4* exhibited pronounced transcript levels in chitin 1% and GlcNAc cultures. SgC chitinases presented lower transcript levels compared to all other chitinase transcripts. *chimaD1* presented higher transcript levels with the mycelia conditions. A HeatMap representation of the transcript levels coupled to a hierarchical clustering of the growth conditions was obtained in order to group conditions with similar expression profiles (Figure 8). Among the analyzed conditions, the cell types (conidia, appressoria, blastospores and growth on tick cuticles) presented lower relative transcript levels, and the mycelia cultures with MCc, chitin 1% and GlcNAc 0.25% presented the most elevated levels of transcripts. Moreover, a closer proximity among chitin 1%, GlcNAc 0.25% and autolysis conditions could be observed, with which transcript levels were higher.

When chitinase expression in conidia was used as a control condition for the 2^−ΔΔCt^ analysis method (Figure S6A to S6D), all sgB chitinases were shown to be induced by chitin 1%, although at different levels. Chitin provided higher induction for *chimaB1*, *B2*, *B3*, *B4* and *B6.* Despite also being induced by chitin, *chimaB5* was more highly induced in complete medium, and *chimaB7* was more highly induced by the two conditions most related to infection: tick cuticles and blastospores. In general, excluding *chimaB7*, chitinases in this subgroup presented lower transcript levels with blastospore, appressoria and tick cuticle conditions compared to transcript levels with conidia. Conversely, chitin in the culture medium was not as strong an inducer of sgA chitinases as it was for sgB. Only three out of nine chitinases of sgA displayed higher transcript levels in the presence of chitin (*chimaA1*, *A6* and *A8)*. *ChimaA5* was most strongly induced in appressoria, and *chimaA7* and *A4* were most strongly induced with complete media culture. *ChimaA2* did not show significantly different levels of transcripts between conditions, which is a pattern previously reported regarding its orthologous gene chi18-3 in *T. reesei*. The autolysis condition did not show any significant induction of sgA chitinases, but seemed to induce *chimaB3* and *C2* gene expression. *ChimaC1* and *C4* showed higher transcript levels with complete media culture, and *chimaD1* and *MaEng18B* showed higher transcript levels with GlcNAc culture.

## Discussion

The relevance of chitinolytic enzymes in fungi is reflected in the number of members included in this multigene family, which is represented as one of the largest family groups among glycoside hydrolases in fungal genomes [60]. Fungal genomes contain diverse multigene families, which are multiple genes with a common origin, encoding similar or identical products that may exhibit the same function or may have diverged to acquire other functions [61]. Therefore, understanding the specific functions of each member of a multigene family, as well as comparing genes among fungal species, provides important information for characterizing gene families. Using birth and death evolutionary model, Karlsson & Stenlid [50] showed that the fungal chitinase family evolved non-randomly, and they were able to identify fungal lineages with expansions and contractions in the number of chitinase gene members.

Thus far, there have been no detailed studies correlating subgroup categorization, phylogenetic inferences and expression data for *M. anisopliae* GH18 genes. A total of 24 GH18 genes, all containing the characteristic GH18 domain, are present in the *M. anisopliae* genome and were detected by a genomic survey using amino acid chitinase sequences from *Metarhizium* spp., *T. reesei* and other fungi as queries in tBLASTn screenings. These genes had detectable transcripts, validating their functionality. GH18 genes and their coding sequences were analyzed in detail by identifying their characteristic properties. Twenty out of 24 GH18 genes belong to subgroups A, B and C, as previously identified for *T. reesei* chitinases, but four *M. anisopliae* genes are grouped into a separated branch. Therefore, we propose to classify them as subgroups D and E. The characteristic properties from the predicted GH18 proteins and their gene structures were detected, and this multigene family from *M. anisopliae* exhibited a range of variation in the aspects considered.

Isoelectric point prediction analysis showed that most of the potential chitinases and ENGases are acidic enzymes (Table S3). According to Pinto et al. [62], *M. anisopliae* acidic enzymes can cleave only GlcNAc oligomers containing more than four residues, which classify them as endochitinases, while basic isozymes act as exochitinases. The high number of acidic enzymes is in agreement with previous studies, in which only one out of eighteen *T. reesei* chitinases and one out of fifteen *Chaetomium globosum* chitinases were predicted to be alkaline enzymes [17], [63]. In *M. anisopliae*, four GH18 proteins were predicted to be alkaline: ChiMaA2, A6, B7 and MaEng18A. A glycol chitin containing SDS-PAGE [33], [64] also resulted in multiple overlapping degradation bands co-localized between 45 and 66 KDa, which is in accordance with the number of predicted acidic chitinases. Although most of these enzymes have molecular masses below this range, some post-translational modifications may alter the estimated MW in SDS-PAGE. Indeed, *T. reesei* chitinases showed glycosylation modifications that may influence the migration pattern and cause distortions in SDS-PAGE [65]. The predicted *M. anisopliae* chitinase glycosylation sites obtained from the *in silico* analysis (an average of 3,4 sites of glycosylation/protein) corroborates the previous experimentally demonstrated band pattern. Because most of the *M. anisopliae* chitinase and ENGase sequences are predicted to have a signal peptide sequence (19/24), this indicates that the secretory pathway is very important for this class of enzymes, as already described for chitinases from other fungi [4]. Moreover, it was possible to detect regions recognized by non-classical secretion pathways in three out of five chitinases that do not contain a signal peptide. ChiMaA4 and C2 are the only chitinases to which it was not possible to assign any secretory characteristic. Only three *M. anisopliae* chitinases were experimentally identified thus far in culture supernatants: CHIT42 (ChiMaA1), CHI2 (ChiMaB1) and CHIT30 (ChiMaB2) [25], [66]. Santi *et al.* [67] detected chitinase activity in *M. anisopliae* intact conidial extracts, indicating that some chitinases may be localized at the spore surface. Chitinase CHIT42 (ChiMaA1) was also detected at the spore surface by immunoproteomic analysis [68].

The analysis of chitinase gene structure revealed two intron positional patterns. The prevalent position of introns upstream of the catalytic site in chitinase genes matches the strong 5′ bias in the intron positions already described in fungal genomes [69]. The second pattern was the insertion position of intron sequences interrupting and surrounding the catalytic site. The presence of an intron sequence in this position may favor the occurrence of sequence modifications. The high positional conservation of introns shared by ChiMaA5 and A7 agrees with the OrthoMCL analysis of orthologs and paralogs and classifies these two chitinases as paralogous. Because ChiMaB1 exhibited two transcripts characterized by the removal or retention of the second intron [59], we examined the intron content of ChiMaA2 and ChiMaB2, which presented two bands in RT-PCR experiments (Figure 1). There is an in-frame stop codon at the beginning of the third intron on ChiMaA2, and this could lead to a smaller transcript while not altering the composition of domains in this protein as suggested for ChiMaB1. Otherwise, ChiMaB2 presents only one intron, and it is positioned interrupting the chitinase catalytic domain. It also contains an in-frame stop codon at the beginning of the intron that, if considered as the correct stop signal, would produce a smaller protein with no domains. The ChiMaB2 chitinase intron sequence and length remains to be further studied before the exact composition of the observed transcripts can be determined. The post-transcriptional regulation of chitinase genes was also already shown for *T. atroviride*. *M. anisopliae chimaB1* produced two forms of transcripts detected under MCc, GlcNAc, autolysis and blastospore conditions and *chimaA2* and *chimaB2* presented the same occurrence under conditions inducing fungal germ tubes and appressoria. The corresponding *chi18-3* and *chi18-13* orthologs in *T. atroviride* also showed similar transcript patterns [17].

The phylogenetic analyses grouped various chitinase and ENGase orthologs and paralogs that were separated into five distinctive subgroups, A, B, C, D and E. Taking into account the tree containing only *Metarhizium* spp. and *T. reesei*, we could observe the four well-supported clades and the known distribution of subgroups into closely related subgroups A and C and subgroups B and the proposed subgroups D and E. The subgroup A tree reveals the orthology among several fungal species, and most of the *M. anisopliae* sgA chitinases are also present in the *M. robertsii* and *M. acridum* genomes, although *M. robertsii* lacks A3, and *M. acridum* lacks both A6 and A7. It is worth noting that sgA has one pair of paralogous chitinases (ChiMaA5 and A7), with A7 absent in both *M. acridum* and *C. militaris* but present in *M. robertsii*. The presence of this protein in *M. robertsii* suggests that the gene duplication may have occurred before the separation of these two species.

The sgB phylogenetic tree contains ChiMaB1 and B2 (CHI2 and CHIT30), for which mutants were already shown to diminish *M. anisopliae* virulence against *D. peruvianus* [25], [26]. Therefore, if based on their presence, this subgroup may contain other important chitinases related to pathogenesis and potential targets for knockout experiments. The chitinases grouped with ChiMaB7 also have ser/thr-rich regions and are GPI-anchored, like both ChiA from *A. nidulans* [70] and ChiA1 from *A. fumigatus*. The glycosylated and GPI-anchored ChiA from *A. nidulans* is located at hyphal branching sites and hyphal tips and has therefore been proposed to be involved in cell wall remodeling at both cell sites [70]. A quintuple mutant lacking all sgB chitinases was constructed in *A. fumigatus*, but no growth or germination defects were observed, suggesting that this family is not crucially involved in the morphogenesis of this fungus. It was suggested that sgB chitinases in *A. fumigatus* might play a nutritional role during autolysis because a slight growth decrease was detected with that condition [71].

The sgC genes are less represented in *M. anisopliae* (4 genes) than in *M. robertsii* (8 genes) or the teleomorph *C. militaris* (5 genes), and *M. acridum* presents even fewer genes (3). Regarding the four additional sgC chitinases present in *M. robertsii* genome, *M. anisopliae* does not have orthologous sequences for two of these genes but does have interrupted orthologs for the other two genes. These two sequences are believed to represent pseudogenes from sgC chitinases given that, except for the presence of multiple stop codons, these two sequences match those of sgC chitinases (EFZ03350 and EFZ03083) from *M. robertsii*. Pseudogenes are nonfunctional genes generated by nonsense mutations, frameshift mutations or partial nucleotide deletions [72]. The two putative C pseudogenes contain 18 and 5 stop codons interrupting the putative *M. anisopliae* coding sequences when compared to *M. robertsii* sequences. By analyzing the alignments between these sequences, possible frameshift errors in *M. anisopliae* sequencing were discarded because the amino acids interpolated by the stop codons showed identical correspondence to the orthologous sequences from *M. robertsii*. This large correspondence throughout the sequences would not occur in a frameshift error where most of the amino acid composition would be impaired. Consequently, these two pseudogene sequences seem not to be sequencing artifacts but were generated by nonsense mutation. All 24 GH18 sequences are distributed into different contigs except for one of the sgC pseudogene sequences, which is grouped closely to other sgC genes. As observed in *Trichoderma* species, sgC proteins have large dissimilarities outside the GH18 and CBM domains, and the ORFs automatically predicted by the genome annotation were incorrect outside these conserved regions, so their sequences were analyzed in deeper detail. We could observe that some of the sgC chitinase sequences available on the NCBI databases from both *M. robertsii* and *M. acridum* genomes have discrepancies in some regions. The protein sequences EFY97746 and EFY97747 from *M. robertsii* (*M. acridum* sequences EFZ86352 and EFZ86353) correspond to only one *M. anisopliae* sgC protein (ChiMaC3) and lack a region between these sequences. Chitinases from this subgroup are better studied in *A. nidulans* and in mycoparasitic *Trichoderma* species [24], [30]. The number of chitinase members in this genus is higher than in other fungal genomes, whereas *T. atroviride* has 9 sgC genes, and *T. virens* has 15 sgC genes, indicating that *M. anisopliae* most likely did not exhibit an expansion like those observed in *Trichoderma*, *Gibberella* and *Uncinocarpus* spp. Moreover, the two putative pseudogenes also indicate that expansion was not important for *M. anisopliae*.

The phylogenetic tree from the proposed subgroups D and E is divided into three major clades separating ChiMaD1 from MaEng18A, MaEng18B and MaEng18C. The classification of these genes into different subgroups was considered based on the amino acid alignments and phylogenetic tree construction. The amino acid sequences that compose this group of proteins were sufficiently distinct to separate these sequences from all other chitinase sequences, as shown by the phylogenetic trees in Figures 2 and S3. The sgD ChiMaD1 is orthologous to Chi18-15 from *T. reesei*, which was reported as acquired by a Hypocreales ancestor by horizontal transfer from *Streptomyces* [17]. This protein did not group with any *T. reesei* chitinases in its first description [17], and it was classified as subgroup B5 by Karlsson & Stenlid [18]. ChiMaD1 enzyme probably acts as a chitinase because of the confirmed chitinolytic activity exhibited by the *B. bassiana* Bbchit1 ortholog [73].

Five fungal ENGases from the GH18 family (orthologous with the three *M. anisopliae* ENGases described) were already characterized and their deglycosylation enzymatic activity was confirmed in *T. reesei*, *T. atroviride* and *F. velutipes* [51], [57], [58]. Although firstly assigned to sgB5 our data shows that the branch separation originated by the phylogenetic analysis is a strong and reliable indication that these protein sequences are very different from sgB proteins. Enzymatic assays to evaluate chitinase activity were also performed in *Trichoderma* ENGases and these enzymes do not exhibited chitinolytic activity [51], [57]. If these proteins are indeed not true chitinases and are actually deglycosylation enzymes, it seems even more plausible to group them in a separated subgroup or even family. To be classified in the subgroup E, these sequences had to present a clustering pattern that separates them in a ramification independent from subgroup B. The only domain included in the sgE (ENGases) and the sgD proteins is the GH18 domain, and there are no sgD or sgE proteins with CBM18, CBM1 or CBM50 domains that are characteristic of subgroups B or C. SgD and sgE proteins are also differentiated by the absence of introns in all members from this subgroup.

The transcription analyses from the chitinase genes validated the predicted sequences, and their relative expression displayed variable transcript profiles under several conditions. The transcription profile of 23 chitinases was examined in *M. anisopliae* at different stages of development: conidia, mycelia (culture medium added of either chitin, GlcNAc, glucose or induced autolysis), appressoria, blastospores and an infection condition (cultures on tick cuticles). The detection of transcripts of each of the 23 GH18 genes in at least one of these conditions validates our gene predictions from the annotated genome. The relative expression levels of chitinases in the three cell types tested (conidia, appressoria and blastospores) were less representative than in the growth conditions in which mycelium was obtained. This lower representativeness may be related to the diminished metabolic activity of resting cells such as conidia. Additionally, blastospores are cell types found in host hemolymph, and at this stage, the fungus has already transposed the chitinous exoskeleton and is using trehalose as a carbon source, being chitinase expression not required. In the appressoria induction condition, the low chitinase transcript representativeness was not expected because the appressorium is a specialized penetration structure that helps to dissolve the host chitinous exoskeleton. These cells use enzyme secretion and physical pressure to mediate penetration but, before penetrating, the appressoria must promote adhesion. To adhere to hydrophobic surfaces, the fungal cell releases adhesion proteins (adhesins and other proteins from the extracellular matrix). It may be that the appressoria cells induced over glass coverslips are at a stage of adhesion were the secretion of chitinases had not yet occurred. In fact, it is known that chitin synthases are essential for promoting appressorium formation because a mutant for one of these genes, *chs7* from *M. oryzae*, was unable to form appressoria on artificial hydrophobic surfaces [74]. The cell wall from the appressoria must be rigid enough and dense enough with chitin fibers to support the process of high turgor generation. As observed in *Colletotrichum graminicola* appressoria, the use of the Nikkomycin Z chitin synthase inhibitor impaired the ability to withstand the necessary turgor pressure [75]. MCc growth cultures, which contain glucose as carbon source, are regarded as a repression condition for chitinase gene expression. Many transcript levels from all chitinase subgroups were high with this condition as much as with the GlcNAc 0.25% and chitin 1% induction cultures. The chitinase expression levels observed in glucose may be due to active fungal growth and the production of mycelial mass that requires chitinases to remodel the fungal cell wall (hyphal branching). As a general overview, there was no common expression profile for all chitinase genes, even when considering members within the same chitinase subgroup.

These differential expression profiles indicate an absence of a common induction/repression expression pattern attainable by all GH18 family members in *M. anisopliae*, suggesting that they may not have totally redundant roles. Redundancy is the most common explanation for multigene families. However, the diversity of the expression profiles may indicate different functions. The importance of individual chitinases has been suggested by our previous results, showing that the knockout of a single chitinase gene (*chimaB1* or *chimaB2*) significantly affects insect infection by *M. anisopliae* [25], [26]. Chitinases A5 and B7 represent good candidates for a more detailed analysis involving *M. anisopliae* virulence against its hosts because those two genes were the most expressed in conditions related to infection.

## Supporting Information

Figure S1 ***M. anisopliae*** **cell types analyzed in this work.** A) *M. anisopliae* appressoria induced over glass coverslips; B) *M. anisopliae* blastospore induction; C) *M. anisopliae* growth over *R. microplus* cuticle.
(DOCX)

Figure S2 **Evolutionary relationships of** ***M. anisopliae*** **,** ***M. robertsii*** **,** ***M. acridum*** **and** ***Cordyceps militaris*** **.** The evolutionary history was inferred using the Neighbor-Joining method conducted in MEGA6 software. The percentage of replicate trees in which the associated taxa clustered together in the bootstrap test (1000 replicates) is shown next to the branches. The tree is drawn to scale, with branch lengths in the same units as those of the evolutionary distances used to infer the phylogenetic tree. The analysis involved nucleotide sequences of the 5′region of the *tef-1-alpha* gene.
(DOCX)

Figure S3 **Categorization of 24 GH18 proteins in the** ***M. anisopliae*** **genome.** *Trichoderma reesei (Hypocrea jecorina)* chitinase amino acid sequences were obtained from the NCBI databases. The three previously described chitinase subgroups (A, B and C) and the two proposed novel subgroups (D and E) are depicted. The Neighbor-Joining (1000 bootstraps) phylogenetic tree was constructed using Mega 6 after ClustalW alignment. The scale bar indicates the genetic distance, which is proportional to the number of amino acid substitutions.
(DOCX)

Figure S4 **Presence of the conserved domains S/A/MxGG and DxxDxDxE in** ***M. anisopliae*** **predicted chitinases and ENGases.** Amino acid sequences were aligned at ClustalX, amino acid background colors follow Clustal default definition. * - indicates 100% conserved residues.
(DOCX)

Figure S5 **Conservation of intron positions on sgA paralogous chitinases A5 and A7.**
(DOCX)

Figure S6 **Relative transcript levels analysis using 2^−ΔΔCt^ method and conidia as the control condition.** (S6A to S6D) Relative transcript expression levels of the GH18 genes from subgroups A, B, C, D and E.
(DOCX)

Table S1 **Chitinase survey of the** ***M. anisopliae*** **genome: fungi, number of chitinase sequences and source.**
(DOCX)

Table S2 **Oligonucleotide sequences used for RT-PCR and qPCR experiments.**
(DOCX)

Table S3 **Additional properties of the 24** ***M. anisopliae*** **GH18 proteins.**
(DOCX)
