# Supplementary material for: Genomic Analyses and Transcriptional Profiles of the Glycoside Hydrolase Family 18 Genes of the Entomopathogenic Fungus Metarhizium anisopliae
Source: PLoS One. 2014 Sep 18;9(9):e107864. doi: 10.1371/journal.pone.0107864 (PMC4169460; doi:10.1371/journal.pone.0107864)
Supplement: Figure S1 — M. anisopliae cell types analyzed in this work. A) M. anisopliae appressoria induced over glass coverslips; B) M. anisopliae blastospore induction; C) M. anisopliae growth over R. microplus cuticle. (DOCX) [file pone.0107864.s001.docx]

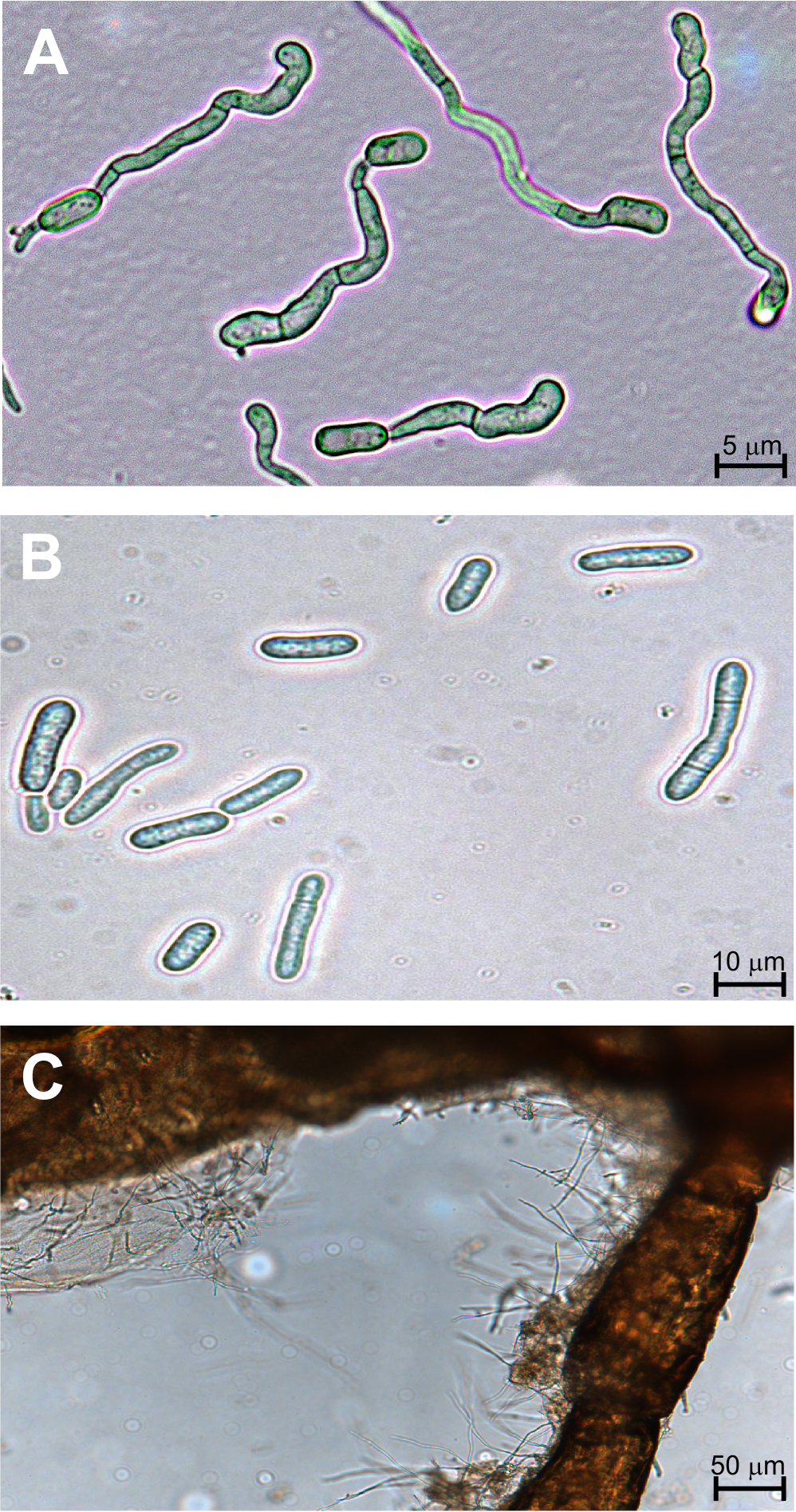


**Figure S1**. *M. anisopliae* cell types analyzed in this work. A) *M. anisopliae* appressoria induced over glass coverslips; B) *M. anisopliae* blastospore induction; C) *M. anisopliae* growth over *R. microplus* cuticle.
