# Supplementary material for: Genomic Analyses and Transcriptional Profiles of the Glycoside Hydrolase Family 18 Genes of the Entomopathogenic Fungus Metarhizium anisopliae
Source: PLoS One. 2014 Sep 18;9(9):e107864. doi: 10.1371/journal.pone.0107864 (PMC4169460; doi:10.1371/journal.pone.0107864)
Supplement: Figure S2 — Evolutionary relationships of M. anisopliae , M. robertsii , M. acridum and Cordyceps militaris . The evolutionary history was inferred using the Neighbor-Joining method conducted in MEGA6 software. The percentage of replicate trees in which the associated taxa clustered together in the bootstrap test (1000 replicates) is shown next to the branches. The tree is drawn to scale, with branch lengths in the same units as those of the evolutionary distances used to infer the phylogenetic tree. The analysis involved nucleotide sequences of the 5′region of the tef-1-alpha gene. (DOCX) [file pone.0107864.s002.docx]

**Figure S2. Evolutionary relationships of *M. anisopliae*, *M. robertsii*, *M. acridum* and *Cordyceps militaris*.** The evolutionary history was inferred using the Neighbor-Joining method conducted in MEGA6 software. The percentage of replicate trees in which the associated taxa clustered together in the bootstrap test (1000 replicates) is shown next to the branches. The tree is drawn to scale, with branch lengths in the same units as those of the evolutionary distances used to infer the phylogenetic tree. The analysis involved nucleotide sequences of the 5´region of the *tef-1-alpha* gene.
