# Supplementary material for: Genomic Analyses and Transcriptional Profiles of the Glycoside Hydrolase Family 18 Genes of the Entomopathogenic Fungus Metarhizium anisopliae
Source: PLoS One. 2014 Sep 18;9(9):e107864. doi: 10.1371/journal.pone.0107864 (PMC4169460; doi:10.1371/journal.pone.0107864)
Supplement: Figure S3 — Categorization of 24 GH18 proteins in the M. anisopliae genome. Trichoderma reesei (Hypocrea jecorina) chitinase amino acid sequences were obtained from the NCBI databases. The three previously described chitinase subgroups (A, B and C) and the two proposed novel subgroups (D and E) are depicted. The Neighbor-Joining (1000 bootstraps) phylogenetic tree was constructed using Mega 6 after ClustalW alignment. The scale bar indicates the genetic distance, which is proportional to the number of amino acid substitutions. (DOCX) [file pone.0107864.s003.docx]

**
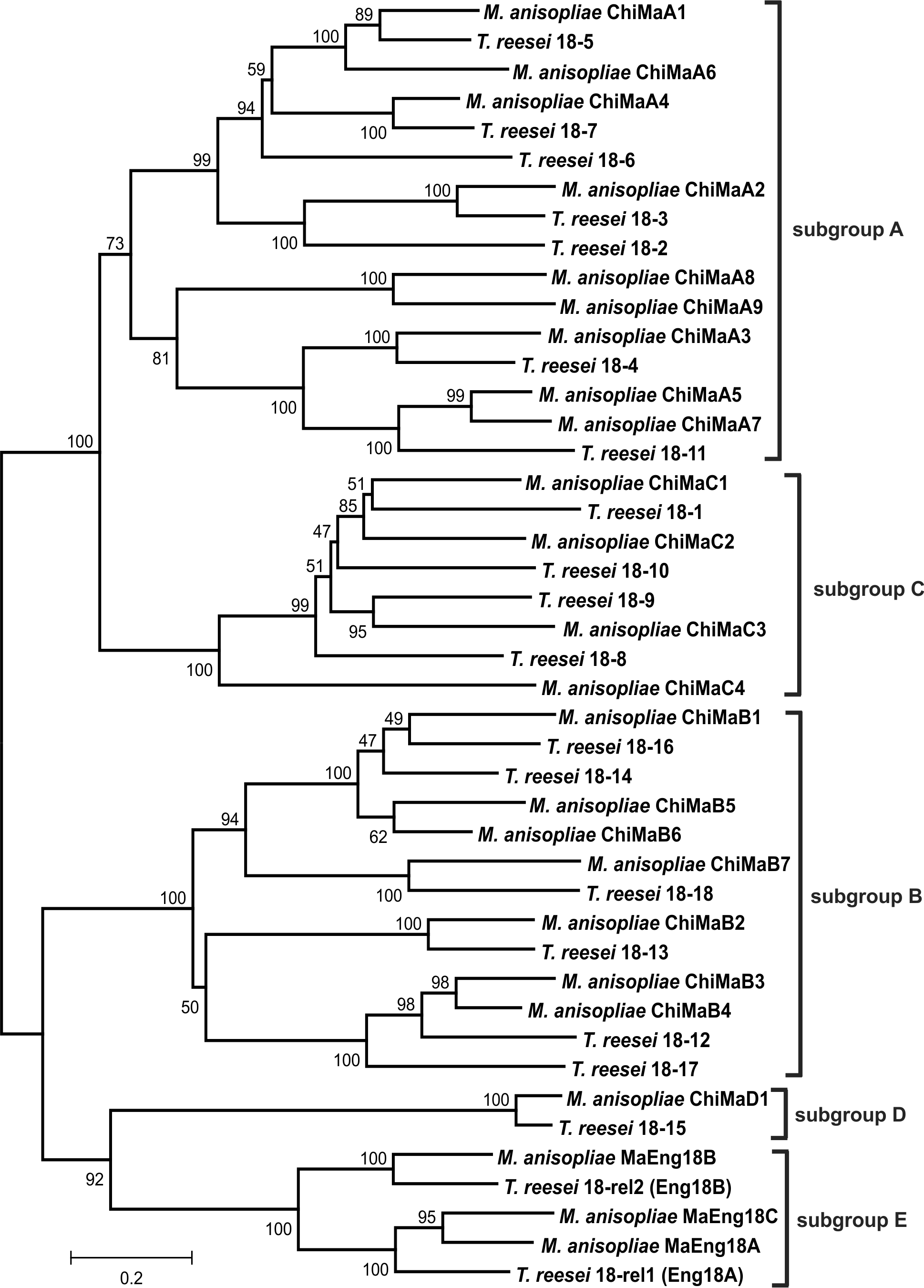
**

**Figure S3.** **Categorization of 24 GH18 proteins in the *M. anisopliae* genome.** *Trichoderma reesei (Hypocrea jecorina)* chitinase amino acid sequences were obtained from the NCBI databases. The three previously described chitinase subgroups (A, B and C) and the two proposed novel subgroups (D and E) are depicted. The Neighbor-Joining (1,000 bootstraps) phylogenetic tree was constructed using Mega 6 after ClustalW alignment. The scale bar indicates the genetic distance, which is proportional to the number of amino acid substitutions.
