# Supplementary material for: Genomic Analyses and Transcriptional Profiles of the Glycoside Hydrolase Family 18 Genes of the Entomopathogenic Fungus Metarhizium anisopliae
Source: PLoS One. 2014 Sep 18;9(9):e107864. doi: 10.1371/journal.pone.0107864 (PMC4169460; doi:10.1371/journal.pone.0107864)
Supplement: Figure S4 — Presence of the conserved domains S/A/MxGG and DxxDxDxE in M. anisopliae predicted chitinases and ENGases. Amino acid sequences were aligned at ClustalX, amino acid background colors follow Clustal default definition. * - indicates 100% conserved residues. (DOCX) [file pone.0107864.s004.docx]

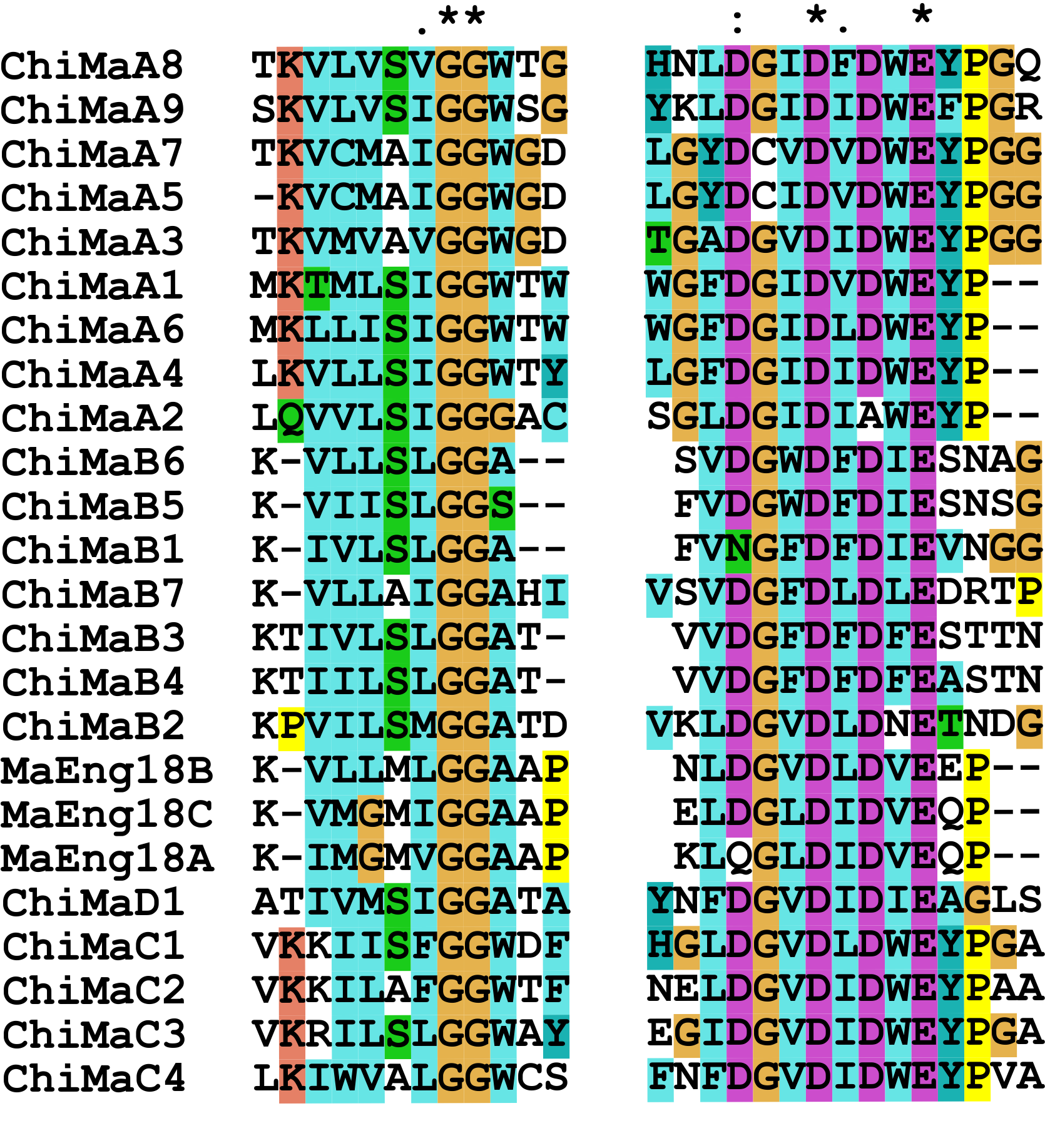


**Figure S4. Presence of the conserved domains S/A/MxGG and DxxDxDxE in *M. anisopliae* predicted chitinases and ENGases**. Amino acid sequences were aligned at ClustalX, amino acid background colors follow Clustal default definition. * - indicates 100% conserved residues.
