# Supplementary figures and images for: Genomic Analyses and Transcriptional Profiles of the Glycoside Hydrolase Family 18 Genes of the Entomopathogenic Fungus Metarhizium anisopliae
Source: PLoS One. 2014 Sep 18;9(9):e107864. doi: 10.1371/journal.pone.0107864 (PMC4169460; doi:10.1371/journal.pone.0107864)

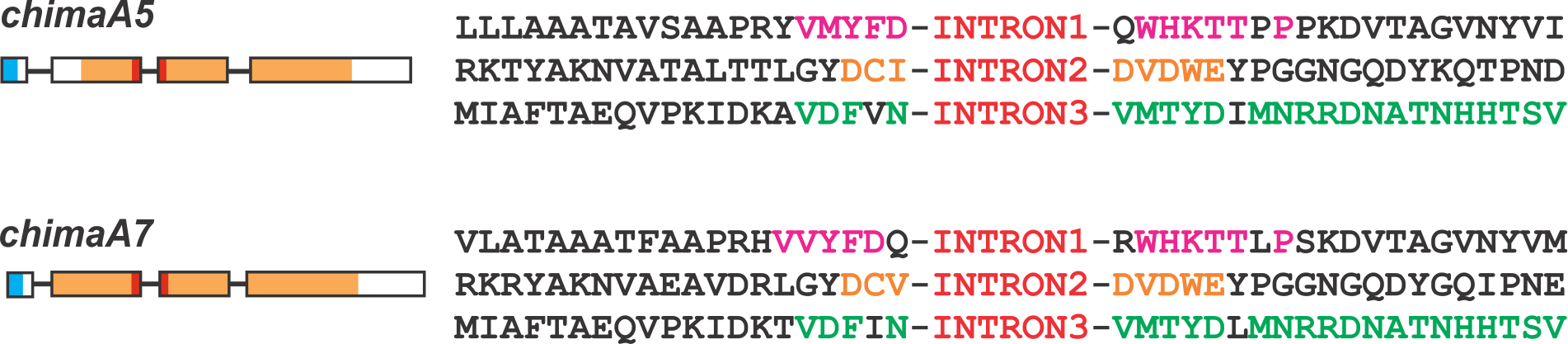


**Figure S5.** Conservation ofintron positions on sgA paralogous chitinases A5 and A7.

Supplement: Figure S5 — Conservation of intron positions on sgA paralogous chitinases A5 and A7. (DOCX) [file pone.0107864.s005.docx]
