# Supplementary material for: Genomic Analyses and Transcriptional Profiles of the Glycoside Hydrolase Family 18 Genes of the Entomopathogenic Fungus Metarhizium anisopliae
Source: PLoS One. 2014 Sep 18;9(9):e107864. doi: 10.1371/journal.pone.0107864 (PMC4169460; doi:10.1371/journal.pone.0107864)
Supplement: Figure S6 — Relative transcript levels analysis using 2−ΔΔCt method and conidia as the control condition. (S6A to S6D) Relative transcript expression levels of the GH18 genes from subgroups A, B, C, D and E. (DOCX) [file pone.0107864.s006.docx]

**
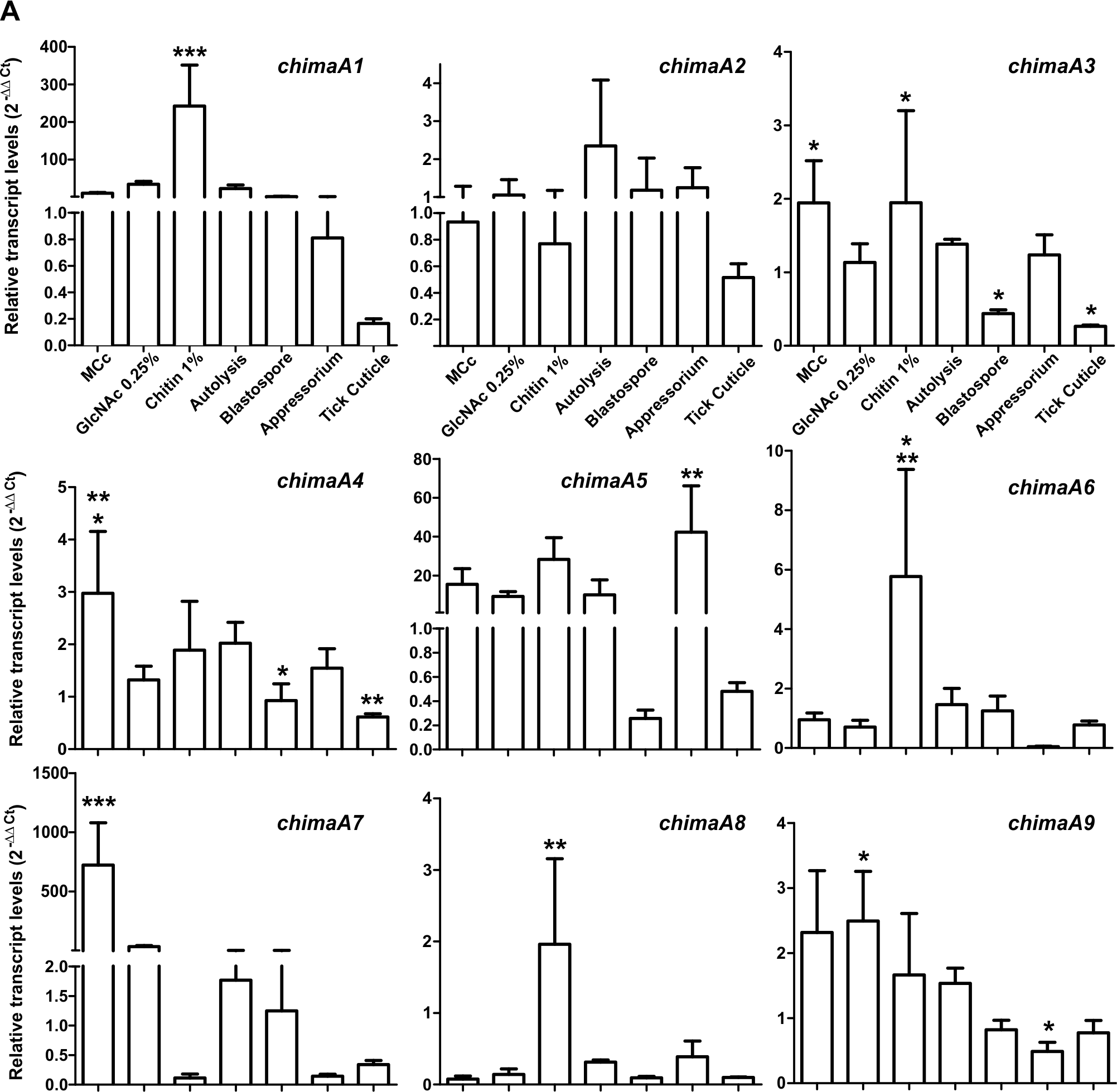
**

**
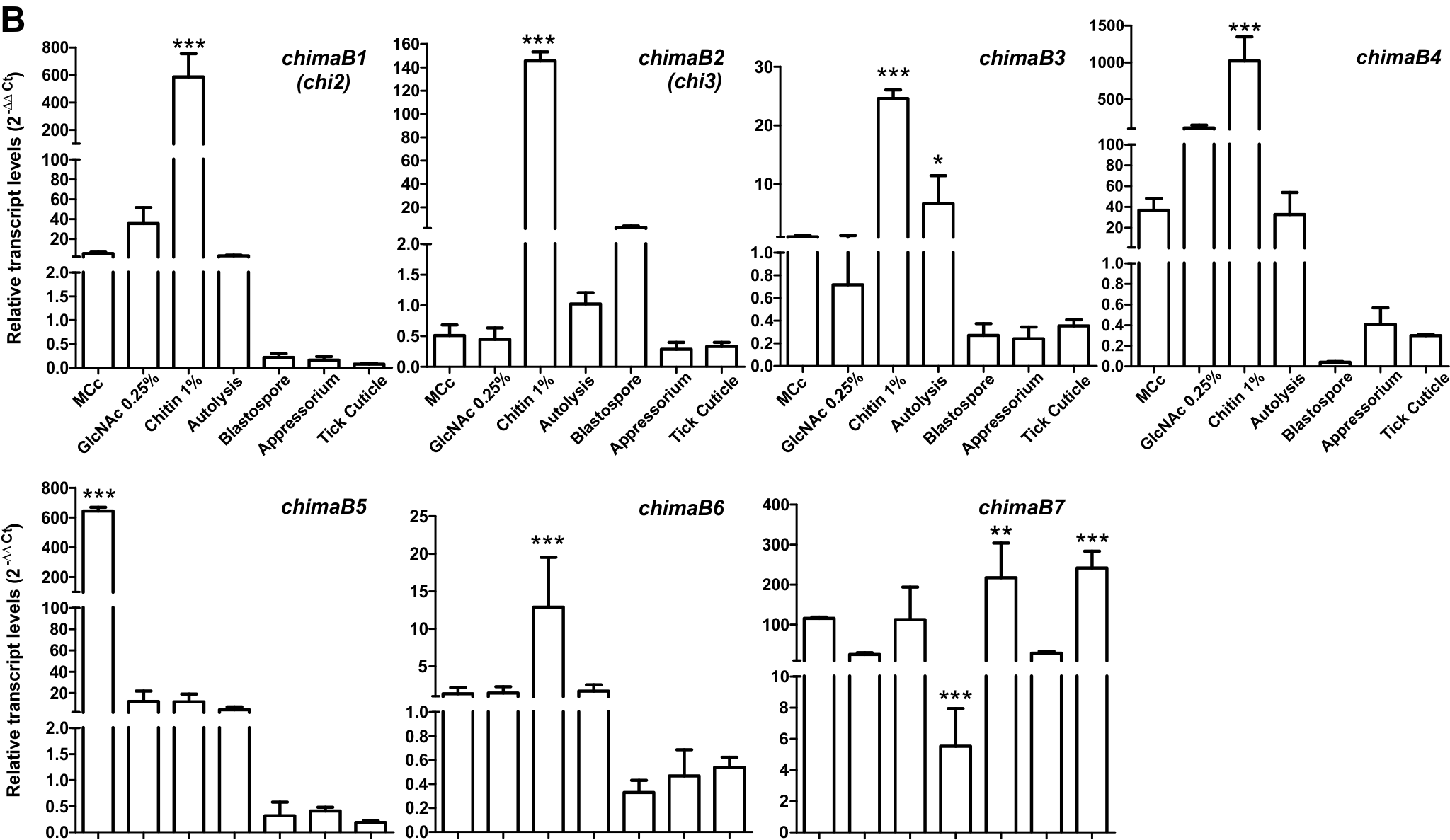
**

**
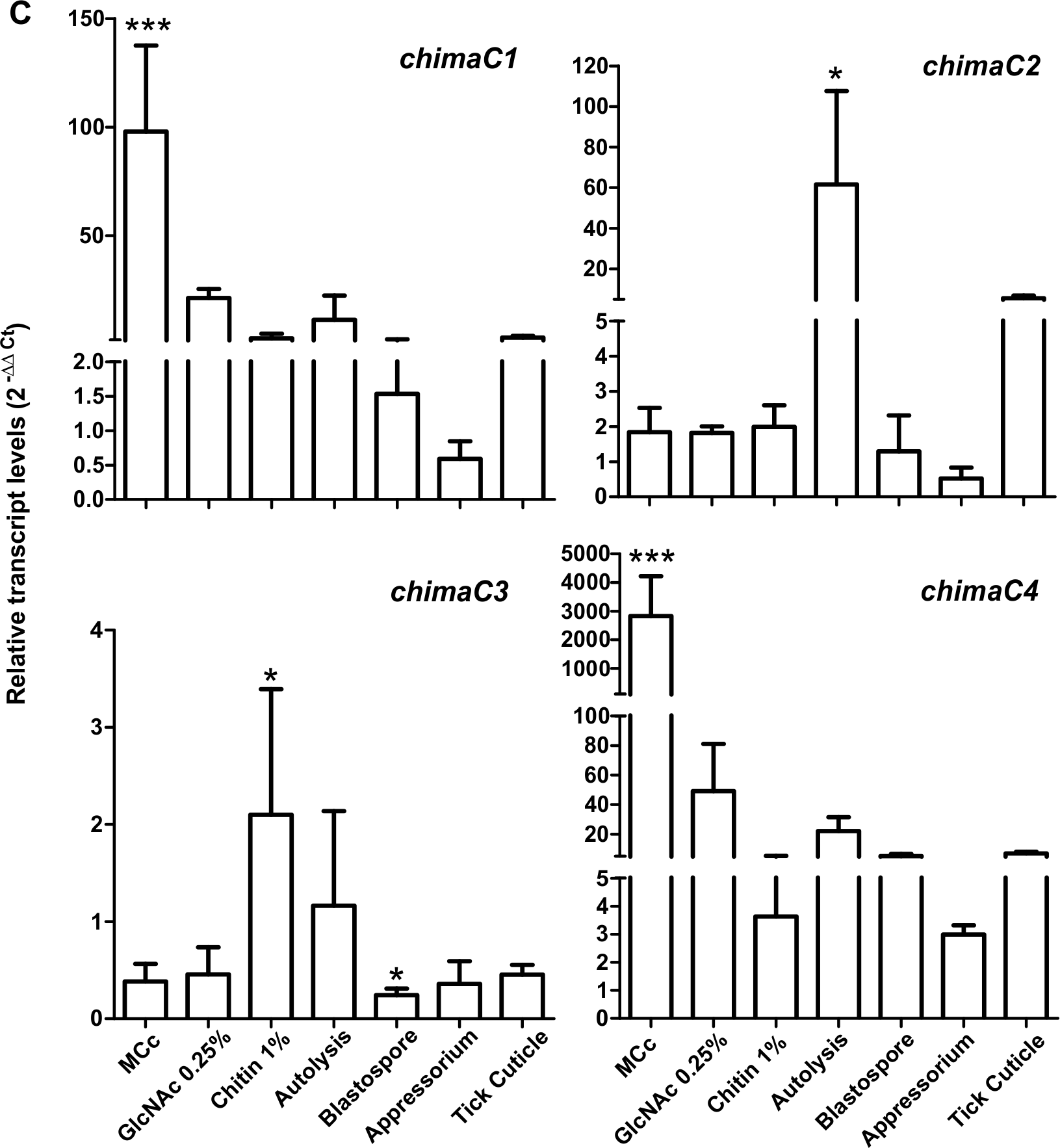
**

**
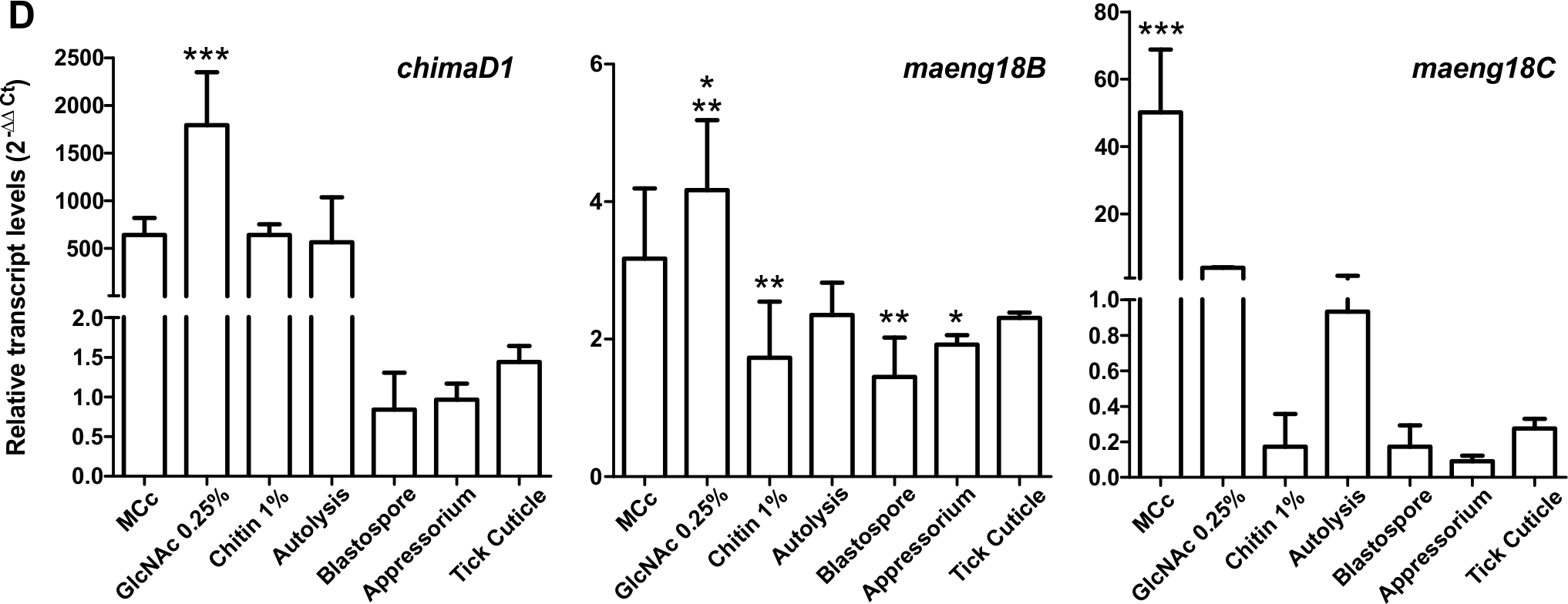
**

**Figure S6 (A-D).** Relative transcript levels analysis using 2^-^**^ΔΔ^**^Ct^ method and conidia as the control condition. S6A to S6D) Relative transcript expression levels of the GH18 genes from subgroups A, B, C, D and E.
