# Supplementary material for: Genomic Analyses and Transcriptional Profiles of the Glycoside Hydrolase Family 18 Genes of the Entomopathogenic Fungus Metarhizium anisopliae
Source: PLoS One. 2014 Sep 18;9(9):e107864. doi: 10.1371/journal.pone.0107864 (PMC4169460; doi:10.1371/journal.pone.0107864)
Supplement: Table S1 — Chitinase survey of the M. anisopliae genome: fungi, number of chitinase sequences and source. (DOCX) [file pone.0107864.s007.docx]

**Table S1.** Chitinase survey of the *M. anisopliae* genome: fungi, number of chitinase sequences and source.

| **Microorganism** | **Number of chitinase genes used** | **Reference or NCBI/BROAD Institute*** |
| --- | --- | --- |
| *Metarhizium robertsii* ARSEF23 | 28 | (Gao et al., 2011), NCBI |
| *M. acridum* CQMa102 | 19 | (Gao et al., 2011), NCBI |
| *Cordyceps militaris* CM01 | 19 | (Zheng et al., 2011), NCBI |
| *H. jecorina* (*T. reesei*) QM6a | 20 | (Seidl et al, 2005), NCBI |
| *Magnaporthe grisea* 70-15 | 15 | BROAD Institute |
| *A. fumigatus* Af293 | 18 | BROAD Institute |
| *A. nidulans* FGSC A4 | 19 | BROAD Institute |
| *Fusarium oxysporum* f. sp. cubense race 1 | 25 | BROAD Institute |
| *Cryptococcus neoformans* var *grubii* H99 | 4 | BROAD Institute |
| *C. gattii* R265 | 4 | BROAD Institute |
| *Histoplasma capsulatum* Nam1 | 10 | BROAD Institute |
| *Paracoccidioides brasiliensis* Pb01 and Pb18 | 5 | BROAD Institute |

*Sequences were from the BROAD Institute and NCBI databases.
