# Supplementary material for: Genomic Analyses and Transcriptional Profiles of the Glycoside Hydrolase Family 18 Genes of the Entomopathogenic Fungus Metarhizium anisopliae
Source: PLoS One. 2014 Sep 18;9(9):e107864. doi: 10.1371/journal.pone.0107864 (PMC4169460; doi:10.1371/journal.pone.0107864)
Supplement: Table S2 — Oligonucleotide sequences used for RT-PCR and qPCR experiments. (DOCX) [file pone.0107864.s008.docx]

**Table S2.** Oligonucleotide sequences used for RT-PCR and qPCR experiments.

| **Primer name** | **Forward/Reverse (5’-3’)** | **Efficiency*** |  |
| --- | --- | --- | --- |
| ChiMaA1 | atgccgtcgttatttgctcagtc/CCACGTCCACCCTCCAATAGA | - |  |
| ChiMaA2 | aaggggacactcctgccatg/CGGCAGTCAGAATATAGCGATCCT | - |  |
| ChiMaA3 | gaaatttcaccacgaaacgaaggtca/CGCCTGTTCAACAAGTCATAGGTCA | - |  |
| ChiMaA4 | atgagtaacgacgggtaccgaac/TCTTGAAATTGGCACTGTAGGTCC | - |  |
| ChiMaA5 | atgcgttcatgcatgtttctg/TCACAGTGACTTGACGCCAGCTTG | - |  |
| ChiMaA6 | atgccgtctctcttcgcttc/CGATGGAGATGAGCAGCTTCA | - |  |
| ChiMaA7 | atgaccgcatttgccccgtcaacggc/GTCTCGGCGATTCATTAAATCGTAG | - |  |
| ChiMaA8 | ATGGCGCCGCTCCTCCACACG/TCAGAGCGGCCCCTTGACGTTG | - |  |
| ChiMaA9 | atgcgtttcgaatcaacactctg/TCACTTGGACCCAAACTTCTTG | - |  |
| ChiMaB1 | ggcggcggtacgatcgaaaataac/ACACATTGGTAAGGGGGAAC | - |  |
| ChiMaB2 | gcagctccctcgtgaagtccat/TGTTGCAGTTGCCGTTGTTG | - |  |
| ChiMaB3 | atgtcttcttctttctggtctttggc/GTTGCACCACCGAGTGACAAGAC | - |  |
| ChiMaB4 | atgacgtttcccgtccagac/CCGCCAAGAGAGAGAATGATTG | - |  |
| ChimaB5 | atgggacaacagagcgtcgcctac/TTAGCAAATCTTACCATTTGTC | - |  |
| ChiMaB6 | atgccctccttcagcaccttc/CTAGCAAGCGCTCGCATCAAC | - |  |
| ChiMaB7 | atgtctttgtcattaggtcttg/ACAGCTTGGCGTTCTTGCTCT | - |  |
| ChiMaC1 | ttcggacgacttctgtattgaatcc/TCAGACCGATTGGCTGCATTTC | - |  |
| ChiMaC2 | cttaatccatccaccaaaca/CTCCATCATTATCGTCGTCG | - |  |
| ChiMaC3 | aaggaacttagaagtctggaataaggg/GCTAAGCCACCAATTAAAGG | - |  |
| ChiMaC4 | atgaaggctcttgtggccttcac/CAGCCAGTTTCGGTTTGATGGATC | - |  |
| ChiMaD1 | atggcgccgctcctccacac/TCAGAGCCGCCCCTTGACGTTG | - |  |
| MaEng18B | atgccacccataccgccgcac/CTATTCGTGGTCTGTGCCATC | - |  |
| MaEng18C | atgttcagatcattatatttgctc/TTATATGCCGCCGCCAAGTTG | - |  |
| chiA1qPCR | GCGAAGTCGGCGGTCACTAT/GCGTATGCGTCGAGTTCATCC | 1,95 |  |
| chiA2qPCR | GCGTCGCCCATTTGATGTCTCA/ATGTGCCATCATTACCGCCACC | 1,86 |  |
| chiA3qPCR | ACGAAGGTCATGGTCGCTGTTG/TCCGCACCAGTCAGGTCAATCA | 1,89 |  |
| chiA4qPCR | GCAACAACGCTTCCACGCCATT/ACCTTCTCCCACGCCCTGAAAG | 1,87 |  |
| chiA5qPCR | CAGGTCCGGGCTCTGTTTGAT/TACGTCTTGCGCGTGGTCTC | 1,89 |  |
| chiA6qPCR | GCCGTCTCTCTTCGCTTCTTCA/GCTGGAAGTTGGTGCCATTGTG | 2,00 |  |
| chiA7qPCR | CCGACGGCTCCGATACCTATCT/GTAGACCCGTCCCAGAACCACT | 2,07 |  |
| chiA8qPCR | ATTACGCTCGCCGTGCTCAC/AATGAACGCTGTGTTGCCTTGC | 1,80 |  |
| chiA9qPCR | ATCAACACTCTGGACAGCACTG/GGGAATCGCAAAGGCAACGT | 2,05 |  |
| chiB1qPCR | ctggacctcctttatcgccgac/atgatgtggagcgcgggattt | 1,95 |  |
| chiB2qPCR | gacacaagctcaccgtcta/CCGCCGAGGTTGCCGATACT | 1,93 |  |
| chiB3qPCR | gcgacaagttccctggcaattc/GCACCACCGAGTGACAAGACT | 1,86 |  |
| chiB4qPCR | tcgccaacgctggtgataattg/CCATTGGTCGCCTGGCAAGA | 1,91 |  |
| chiB5qPCR | gacaacagagcgtcgcctact/AAGGACCACTACGTCGATGCC | 1,87 |  |
| chiB6qPCR | tcgtgggccggtatagtagc/TAGCCAGAATCGCCTTGGACAC | 2,05 |  |
| chiB7qPCR | atacaccacctccgtccacacc/GCAGTCTCCGTGACAGGGCATA | 1,86 |  |
| chiC1qPCR | gcctaccagtctcgccaagt/GCAGATTGTTGTCGGAGCCATA | 2,03 |  |
| chiC2qPCR | agccagagccgtcaactatgag/CGAACCAAGCCTCCTGAACCTT | 2,08 |  |
| chiC3qPCR | tgcgtctgcgatgacatgct/GCCAAGAGAACAATGCCTGACC | 1,88 |  |
| chiC4qPCR | agcctcgcagtgttgtggaatg/CGAACCAGGAACTCGGCATAGT | 2,06 |  |
| chiD1qPCR | gtggtggctcaacatgcagtac/GCCCTTTATCCAAGCAGTCGGT | 2,03 |  |
| eng18BqPCR | cgccactacgacagctcatc/TCGAAGCCGCTCAGGTTCTT | 1,95 |  |
| eng18CqPCR | tggtggctggacagctttcttc/ACCTCCAAGGTTCATCCATGCC | 1,88 |  |
| actinqPCR | ACCGTGAGAAGATGACCCAGAT/GAGCAATGGCTGGAGGAAGAG | 2,03 |  |

*The mean qPCR primer efficiencies were calculated using the LinRegPCR software application.
