# Supplementary material for: Genomic Analyses and Transcriptional Profiles of the Glycoside Hydrolase Family 18 Genes of the Entomopathogenic Fungus Metarhizium anisopliae
Source: PLoS One. 2014 Sep 18;9(9):e107864. doi: 10.1371/journal.pone.0107864 (PMC4169460; doi:10.1371/journal.pone.0107864)
Supplement: Table S3 — Additional properties of the 24 M. anisopliae GH18 proteins. (DOCX) [file pone.0107864.s009.docx]

**Table S3.** Additional properties of the 24 *M. anisopliae* GH18 proteins.

| **Identification** | **ORF length (nt)** | **Protein length (aa)** | **Theoretical pI** | **Mature protein theoretical kDa** | **N-Glyco** |
| --- | --- | --- | --- | --- | --- |
| **ChiMaA1 (*chit1*)** | 1525 | 424 | 5.38 | 44.12 **(42)** | 5 |
| ChiMaA2 | 1321 | 359 | 8.19* | 38.60 | - |
| ChiMaA3 | 1306 | 397 | 5.25 | 42.62 | 1 |
| ChiMaA4 | 1301 | 395 | 4.91* | 44.18 | 3 |
| ChiMaA5 | 1309 | 369 | 5.60 | 38.78 | 2 |
| ChiMaA6 | 1461 | 420 | 8.82 | 43.49 | 9 |
| ChiMaA7 | 1289 | 371 | 5.09 | 39.36 | 1 |
| ChiMaA8 | 1209 | 402 | 4.53 | 41.67 | 5 |
| ChiMaA9 | 1188 | 395 | 6.07 | 41.20 | 3 |
| **ChiMaB1 (*chi2*)** | 1544 | 419 | 4.84 | 41.96 **(42)** | 3 |
| **ChiMaB2 (*chi3*)** | 1011 | 317 | 5.11 | 32.16 **(30 e 32.4)** | 2 |
| ChiMaB3 | 1304 | 329 | 6.18 | 32.71 | - |
| ChiMaB4 | 1442 | 421 | 4.95 | 42.95 | 1 |
| ChiMaB5 | 921 | 306 | 5.14 | 33.02 | - |
| ChiMaB6 | 1023 | 340 | 4.82 | 33.30 | 2 |
| ChiMaB7 | 2525 | 793 | 7.91 | 80.63 | 4 |
| ChiMaC1 | 4350 | 1370 | 5.10 | 148.23 | 6 |
| ChiMaC2 | 4111 | 1159 | 6.52* | 127.58 | - |
| ChiMaC3 | 5101 | 1556 | 6.64 | 168.76 | 7 |
| ChiMaC4 | 3589 | 1145 | 6.10 | 125.68 | 3 |
| ChiMaD1 | 1038 | 345 | 5.38 | 33.81 | 1 |
| MaEng18A | 1071 | 356 | 8.34 | 38.57 | 3 |
| MaEng18B | 1017 | 338 | 5.57* | 37.58 | - |
| MaEng18C | 978 | 325 | 5.89 | 34.33 | 3 |

Chitinase and ENGase nucleotide (nt) and amino acid (aa) lengths, predicted molecular mass (kDa), predicted isoelectric point (pI) and predicted post-transcriptional N-glycosylation (Glyco) are indicated. Previously studied chitinases are highlighted in bold. * indicates absence of predicted signal peptide. At mature protein kDa column, the experimentally observed molecular mass is indicated between parentheses.
